# Changes in Metabolically Active Bacterial Community during Rumen Development, and Their Alteration by Rhubarb Root Powder Revealed by 16S rRNA Amplicon Sequencing

**DOI:** 10.3389/fmicb.2017.00159

**Published:** 2017-02-07

**Authors:** Zuo Wang, Chijioke Elekwachi, Jinzhen Jiao, Min Wang, Shaoxun Tang, Chuanshe Zhou, Zhiliang Tan, Robert J. Forster

**Affiliations:** ^1^Key Laboratory for Agro-Ecological Processes in Subtropical Region, Hunan Research Center of Livestock and Poultry Sciences, South-Central Experimental Station of Animal Nutrition and Feed Science in Ministry of Agriculture, Institute of Subtropical Agriculture, Chinese Academy of SciencesChangsha, China; ^2^University of Chinese Academy of SciencesBeijing, China; ^3^Lethbridge Research and Development Centre, Agriculture and Agri-Food CanadaLethbridge, AB, Canada

**Keywords:** rumen development, bacterial colonization, microbial ecology, microbial programming, early weaning, black goats, rhubarb

## Abstract

The objective of this present study was to explore the initial establishment of metabolically active bacteria and subsequent evolution in four fractions: rumen solid-phase (RS), liquid-phase (RL), protozoa-associated (RP), and epithelium-associated (RE) through early weaning and supplementing rhubarb root powder in 7 different age groups (1, 10, 20, 38, 41, 50, and 60 d) during rumen development. Results of the 16S rRNA sequencing based on RNA isolated from the four fractions revealed that the potentially active bacterial microbiota in four fractions were dominated by the phyla *Proteobacteria, Firmicutes*, and *Bacteroidetes* regardless of different ages. An age-dependent increment of Chao 1 richness was observed in the fractions of RL and RE. The principal coordinate analysis (PCoA) indicated that samples in four fractions all clustered based on different age groups, and the structure of the bacterial community in RE was distinct from those in other three fractions. The abundances of *Proteobacteria* decreased significantly (*P* < 0.05) with age, while increases in the abundances of *Firmicutes* and *Bacteroidetes* were noted. At the genus level, the abundance of the predominant genus *Mannheimia* in the *Proteobacteria* phylum decreased significantly (*P* < 0.05) after 1 d, while the genera *Quinella, Prevotella, Fretibacterium, Ruminococcus, Lachnospiraceae NK3A20 group*, and *Atopobium* underwent different manners of increases and dominated the bacterial microbiota across four fractions. Variations of the distributions of some specific bacterial genera across fractions were observed, and supplementation of rhubarb affected the relative abundance of various genera of bacteria.

## Introduction

The complex rumen ecosystem harbors various prokaryotic (bacteria and archaea) and eukaryotic (protozoa and fungi) microorganisms which symbiotically convert feed stuffs into microbial biomass and fermentation end products that can be utilized by the host (Dehority, 2003; Kong et al., 2010). The ruminal microbiome is characterized by its high population density, wide diversity, and complexity of interactions, and it was suggested that the abundance of various microbial genotypes within the rumen can be significantly bound up with host feed efficiency and diet (Zhou et al., 2010; Carberry et al., 2014). In young ruminants, ingested microbes colonize and establish in a defined and progressive sequence shortly after birth during rumen development (Fonty et al., 1987, 2007; Abecia et al., 2013). During the last few decades, extensive efforts have been taken to explore the relationship between the microbial colonization and rumen development applying different methods (Fonty et al., 1987; Skillman et al., 2004; Jami et al., 2013; Rey et al., 2014; Guzman et al., 2015; Jiao et al., 2015b), as the composition of ruminal microflora directly influences the digestive and metabolic performance of the host animal, and therefore the developing rumen in the newborn ruminants provides a unique opportunity to manipulate the diverse commensal microflora (Yáñez-Ruiz et al., 2015). It has been reported that early dietary experiences of animal have a greater and more lasting effect than those occurring later in life (Distel et al., 1994), which implies the feasibility to regulate the rumen microbial community at the early period of rumen development, i.e., microbial programming. Li et al. (2012) found that the microbiome in the developing rumen of 14 days old calves was responsive to dietary modifications as well as physiological changes in the host. Further studies (Yáñez-Ruiz et al., 2010; Abecia et al., 2014) suggested that it would be possible to promote different microbial populations inhabiting the rumen of the young animal by controlling the feeding management in early life that persisted in later life. However, more research is essential to gain insight into the development of the rumen and its microbiome, and the method (e.g., the alteration of diets and the supplementation of specific additives) and timing to manipulate the ruminal microflora in early life.

Rhubarb (*Rheum* spp.) is a commonly used herb in traditional Chinese medicine to cure indigestion, constipation, and other diseases since ancient times, and it has been characterized with antimicrobial and antitumor properties in previous research (Kang et al., 2008; Kosikowska et al., 2010). Previous *in vitro* and *in vivo* studies reported that rhubarb could modify rumen fermentation by lowering methane production and the acetate: propionate ratio without inhibiting feed intake or the degradation of substrates (Bodas et al., 2008; García-González et al., 2010, 2012; Kim et al., 2016). As all these investigations were focused on mature ruminants and rumen fermentation, it would be promising to explore the effects of rhubarb as feed additive in shaping the rumen bacteria community in early life during rumen development.

Within the rumen ecosystem, bacterial species are considered to have a more important role compared to protozoa and fungi in determining the extent and rate of feed degradation and utilization for the production of microbial protein and VFA, which significantly contributes to the maintenance and to the production of meat and milk of the host ruminant (Miron et al., 2001). Hence, improving the understanding of rumen bacterial ecology and acquiring methods to shape the bacterial community may help increase feed efficiency and enhance production performance of animals. The bacterial community in the rumen can been divided into four classes according to their different spatial locations: (1) free-living bacteria associated with the rumen liquid phase; (2) bacteria associated with feed particles; (3) bacteria associated with rumen epithelium; and (4) bacteria attached to the surface of protozoa or contained as endosymbionts inside the protozoal cells (Koike et al., 2003; Yu and Forster, 2005). Amongst the four bacterial groups, the bacteria associated with feed particles, i.e., the solid-phase bacteria, are the most predominant and occupy up to 75% of the total microbial population, and are estimated to produce up to 90% of the endoglucanase and xylanase activities in the rumen (Wang et al., 2011). In contrast, bacteria associated with the liquid-phase (RL) take up 20 to 30% of the total microbes on high-forage rations (Miron et al., 2001). To our knowledge, the majority of the studies on rumen bacteria had been focused on the solid-phase and/or (RL) bacteria (Skillman et al., 2004; Kong et al., 2010; Wang et al., 2011; Jami et al., 2013; Guzman et al., 2015; Jiao et al., 2015b), while research aimed at the bacterial associated with ruminal protozoa and epithelium was limited despite the fact that the presence/absence of protozoa has been found to relate to the structure of different bacterial communities and different rumen fermentation pattern (Yáñez-Ruiz et al., 2007; Belanche et al., 2015). Moreover, the epimural bacterial community which significantly differs from that of rumen contents may influence the extent of development of the rumen epithelium and the immune function (Malmuthuge et al., 2012, 2014). Therefore, it would be of great significance to comprehensively explore the simultaneous evolution of the bacterial populations associated with these four fractions during rumen development.

Up to now, most of the investigations on the rumen microbiota using 16S rRNA gene sequencing have been conducted based on DNA-derived amplicons (Li et al., 2014; Guzman et al., 2015; Liu et al., 2016; Wang et al., 2016), which could indicate the comprehensive diversity of both the living and inactive microorganisms. Nevertheless, DNA-based studies do not reflect the potential biological activity of the rumen microbial community in real time (Hugoni et al., 2013; Salter et al., 2015). By contrast, RNA-based techniques could help gain insights into the metabolic state of microbes and thus could be used to indicate the most active rumen microorganisms and their metabolic potential (Kang et al., 2013; Li et al., 2016). Lettat and Benchaar (2013) reported that despite the minor disparities in the abundance of different taxa, the RNA-based analysis was more discriminative than the DNA-based counterpart in identifying diet-induced alterations within the microbial community.

In the present study, we aim to deepen the understanding of bacterial colonization during rumen development and offer theoretical foundations for the manipulation of rumen microbiome and fermentation in early life. We used RNA-based 16S rRNA amplicon sequencing to investigate the initial colonization and diversity of metabolically active bacteria in four fractions (i.e., the ruminal solid-phase, RL, protozoa, and epithelium) and the subsequent evolution from 1 to 60 d after birth, and the influences of early weaning and the supplementation of rhubarb on the rumen bacterial population.

## Materials and methods

The experiment was approved by the Animal Care Committee, Institute of Subtropical Agriculture, the Chinese Academy of Sciences, Changsha, China.

### Animals, diets, and management

Forty-five newborn Xiangdong (native breed) black goats (*Capra hircus*) were used in this study and housed in a well-ventilated room with controlled temperature and humidity. The experimental start for each goat was staggered to accommodate differing birth dates. After birth, the goats were left with their dams until weaning. On 1, 10, and 20 d, 8, 7, and 6 goats were slaughtered, respectively. The remaining goats were gradually weaned off goat milk and supplied with *ad libitum* a mixture of fresh grass (*Miscanthus sinensis*, 40% of total dry matter [DM]) and starter concentrate (60% of total DM) from 15 d until they were weaned at 40 d. Four goats were further slaughtered at 38 and 41 d, respectively. Sixteen goats were randomly assigned to two diet treatments: the control diet and the diet supplemented with rhubarb root powder, and then reared separately from the dams after weaning. The rhubarb root powder was purchased from a local herbalist retailer and consisted of the dried and milled rhizomes of *Rheum offcinale* Baill.

The control diet (per kg DM) contained 400 g fresh grass (in DM) and 600 g starter concentrate (in DM), and every 600 g starter concentrate was composed of the following components: 193 g extruded soybean, 69 g whey powder, 100 g maize flour, 109 g fat powder, 80 g soybean meal, 6 g CaCO_3_, 15 g CaHPO_4_, 8 g NaCl, and 20 g premix. Every 1 kg premix contained 2.5 g FeSO_4_•7H_2_O, 0.8 g CuSO_4_•5H_2_O, 3 g MnSO_4_•H_2_O, 5 g ZnSO_4_•H_2_O, 10 mg Na_2_SeO_3_, 40 mg KI, 30 mg CoCl_2_•6H_2_O, 95000 IU vitamin A, 17500 IU vitamin D, and 18000 IU vitamin E. In the control treatment, goats were fed 150 g control diet twice per day at 08.00 and 17.00 h, and four goats were slaughtered before morning feeding separately at 50 and 60 d. In the rhubarb supplemented group, goats were gradually accustomed to the supplementation of rhubarb from 1 week before weaning. Two goats were removed for the reason irrelevant to the experiment, the remaining six goats received 150 g control diet plus 2 g rhubarb root powder per meal, and three goats were slaughtered at 50 and 60 d, respectively. The management of goats and sampling is further illustrated in Figure S1, and the increase of body weight is shown in Figure S2.

### Sample fractionation

After the goats were slaughtered, the rumen was immediately removed for sampling of the four fractions, i.e., the ruminal solid-phase samples (RS), the ruminal (RL) samples (RL), the ruminal protozoa samples (RP), and the ruminal epithelium samples (RE). RS and RL samples were collected and separated using a French press filter (Bodum Inc., Triengen, Switzerland) according to the method described by Kong et al. (2010). To obtain the RP samples a modified method of Leng (1982) was used, briefly 10 mL of rumen fluid was centrifuged at 500 g for 1 min and the protozoal pellet was then rinsed with sterile anaerobic saline solution and collected by centrifugation (500 × g). The rinsing was repeated three times. Three RP samples from each goat were pooled for analysis. For the RE samples, 3 pieces of 2 g (approximately 4 cm^2^) epithelium samples were excised at different sites of the same rumen and washed with sterile saline solution and then combined. As the rumen was underdeveloped and the contents were limited, no RS and RP sample was collected on 1 and 10 d, and only 4 RL samples were collected on 1 d. 5 RS samples and 4 RP samples were collected on 20 d. All the samples were immediately flash-frozen in liquid nitrogen and then stored at −80°C for subsequent use.

### RNA extraction and first-strand cDNA synthesis

To isolate total RNA a modification of the method described by Wang et al. (2011) was used. Briefly, samples were first manually ground into crude powder in liquid nitrogen using a mortar and pestle, and then 2 g of crude powder was, respectively, weighed and further ground for 5 min in liquid nitrogen using a Retsch RM100 grinder (Retsch GmbH, Haan, Germany). After grinding, 0.3 g frozen fine powder was weighed into each 50-mL tube and mixed with 3 mL of Ambion TRIzol reagent (Life Technologies, Carlsbad, USA). Subsequent procedures were conducted in accordance with the method of Wang et al. (2011). After the extraction, an Ambion MEGAclear kit (Life Technologies, Carlsbad, USA) was used to purify the isolated RNA. The RNA concentration and integrity were estimated using an Agilent 2100 bioanalyzer and RNA 6000 Nano kit (Agilent Technologies, Santa Clara, USA). The prokaryotic total RNA nano assay protocol was used, as prokaryotes account for the majority of RNA in rumen contents (Yu and Forster, 2005).

Five hundred nanogram of isolated total RNA from each sample was used to synthesize the first-strand cDNA using an Invitrogen SuperScript III RT kit (Life Technologies, Carlsbad, USA), and the cDNA synthesis reactions were stored at −20°C until further analysis was performed.

### PCR amplification and 16S rRNA amplicon sequencing

The PCR amplification of bacterial 16S rRNA genes was conducted on a Dyad Peltier Thermal Cycler (AL056543, Bio-Rad Laboratories, Hercules, USA) using primers Bact_341F (5′-TATGGTAATTGTACTCCTACGGGNGGCWGCAG-3′) and Bact_806R (5′-AGTCAGTCAGCCGGACTACHVGGGTATCTAAT-3′) (Herlemann et al., 2011; Caporaso et al., 2012). A dual barcode assay adapted for the Illumina MiSeq sequencer (Illumina Inc., San Diego, USA) was used (Table S1). Each primer contained the Illumina adapter sequence, unique barcode, spacer and forward or reverse primer. For each cDNA sample, 20 μL of reaction mix was prepared containing 1 μL cDNA, 1 μL of each barcoded primer (1 μM), 7 μL molecular biology grade H_2_O, and 10 μL KAPA2G Robust Hotstart ReadyMix (Kapa Biosystems, Wilmington, USA). The PCR procedures were as follows: initial denaturation at 95°C for 5 min; 20 cycles of denaturation (95°C, 20 s), annealing (55°C, 15 s) and elongation (72°C, 5 min); and a final 10-min extension at 72°C. Each cDNA sample was amplified in duplicates, and three wells per run served as a negative control for the master mix. After amplification, duplicate PCR products were pooled, and the correct sizes of PCR products and the absence of signal from negative controls were further verified through agarose gel electrophoresis. Quantitation of amplicons was performed in a Synergy HTX Multi-Mode Microplate Reader (model SIAFRM, Bio-Tek Instruments Inc., Winooski, USA) using a Quant-iT dsDNA Assay Kit (Thermo Fisher Scientific, Waltham, USA). The amplicons were pooled in equimolar concentrations and purified using Agencourt AMPure XP beads (Beckman Coulter Inc., Brea, USA) and then further quantified as described above. The amplicon library was combined with 10% PhiX control library and sequenced in the Illumina Miseq (Illumina Inc., San Diego, USA) using a v3 600 cycle (300 cycles per read) paired end kit.

### Bioinformatic analysis

The quality of the raw fastq files were checked with the FastQC program (http://www.bioinformatics.bbsrc.ac.uk/projects/fastqc). Trimmomatic v0.33 (Bolger et al., 2014) was used to trim the raw reads, to remove ambiguous and low quality reads. Reads with average quality score <20 over a 4 bp sliding window and reads with lengths shorter than 36 bp were removed. Merging of the paired-end reads was effected with PEAR v0.9.8 using default options (Zhang et al., 2014). Reads which did not get assembled were discarded. High quality sequence reads from the various samples were then combined into a single dataset and subsequent analysis was carried out using the open-source software package, QIIME V1.8.0 (Caporaso et al., 2010b). This involved picking Operational Taxonomic Units (OTUs), assigning taxonomy, inferring phylogeny, creating OTU tables and computing microbial community diversity indices. The sequences were clustered into OTUs using the *de novo* OTU picking workflow with a 97% similarity threshold. Taxonomic assignment of OTUs was performed by comparing the most abundant “representative sequences” within each OTU to the SILVA v119 database (Yilmaz et al., 2014). To enable calculation of Unifrac distances (Lozupone et al., 2011) and to facilitate downstream diversity analysis the picked OTUs were aligned by PyNAST (Caporaso et al., 2010a) against the core alignment template of SILVA v119, and a phylogenetic tree was built using FastTree (Price et al., 2009). To differentiate the conserved from the non-conserved regions of the alignment and remove sections comprised of only gaps (useful in phylogenetic tree construction) a lanemask file was applied. This was constructed from the SILVA v119 core alignment file using a python script. The alpha (within sample) diversity of the samples was estimated using the Chao1, Shannon and observed_otus indices. The Chao1 index was used to further compare the alpha diversity of the samples. Beta (between sample) diversity of the samples was also computed and visualized with three dimensional PCoA plots generated using the Bray-Curtis dissimilarity index (Bray and Curtis, 1957) and the unweighted UniFrac distances. Information on the summary of sequencing data is displayed in the Table S2. All the sequences in the present study were deposited to the sequence read archive (SRA) of the NCBI database using files generated by Mothur V1.33.3 (Schloss et al., 2009), under the accession number SRP081114.

### Statistical analysis

Before the analysis of relative abundance data at phylum and genus level, the compliance of data with the assumptions of normality and homogeneity of variances were first examined visually through residual plots created by the UNIVARIATE and PLOT procedures (SAS Institute Inc., 2001), and variables that were deemed non-normal were then arcsine transformed. Data of bacterial relative abundances at phylum and genus level were analyzed as a completely randomized design using the PROC MIXED procedure of SAS (SAS Institute Inc., 2001) to test the effect of fractions of samples, the model included fraction, age, and fraction × age as the fixed effects, with individual animal as the experimental unit. To test the effect of age on the relative abundances of bacteria at phylum and genus level, the PROC MIXED procedure of SAS (SAS Institute Inc., 2001) was used, with animal nested within age as the random effect and individual animal as the experimental unit. Linear, quadratic, and cubic effects of age were analyzed using orthogonal polynomial contrasts. To compare the relative abundances between the control diet group and the rhubarb group, the PROC MIXED procedure of SAS (SAS Institute Inc., 2001) was used with a model which included the fixed effects of diet, age and diet × age interaction, with individual animal as the experimental unit. Least squares means are reported throughout the text, and statistical significance was declared at *P* < 0.05.

## Results

### Rumen bacterial community composition in four fractions during rumen development and with supplementation of rhubarb amongst individuals

In total, 32 bacterial phyla were identified throughout the four fractions during rumen development. *Proteobacteria, Firmicutes*, and *Bacteroidetes* were detected as the three dominant phyla, which together took up from 37.2 to 99.7% of the bacterial microbiota across different fractions in individuals (Figure S3). By comparison, the phyla Fibrobacteres, *Spirochaetae, Actinobacteria*, and Synergistetes were less predominant and their total proportion in individuals ranged from 0.2 to 61.2%. The abundances of *Proteobacteria* in all the fractions except RS decreased dramatically as the age increased, while the phylum Synergistetes became more abundant on 50 and 60 d than it was before.

In the control diet group at 50 d, *Proteobacteria* accounted for up to 70.3% of the bacterial community in RE, while its abundance was reduced to as low as 13.1% in RE of the rhubarb treatment group (Figure S4). No noticeable difference of the bacterial community composition was observed between two treatments on 60 d.

According to taxonomic assignment, a total of 30 bacterial genera with >0.5% of relative abundance amongst four fractions throughout rumen development were observed (Figure S5). The proportion of the unclassified bacteria and the genera with abundance <0.5 was considerable and ranged from to 4.6 to 90.5% across different fractions in individuals. In the control diet treatment at 50 d, up to 63.0% of the bacterial community in RE was represented by the *Suttonella* spp. (Figure S6), however its abundance ranged from approximately 0.0–25.1% in RE of the rhubarb treatment. Moreover, in the control diet treatment on 60 d, the abundance of the genus *Quinella* in RL of individuals ranged from 23.0 to 60.0%, while it decreased to as low as 7.0% in RL of the rhubarb treatment.

### Diversity of rumen bacteria in four fractions during rumen development and with rhubarb treatment

**Figure 1:**
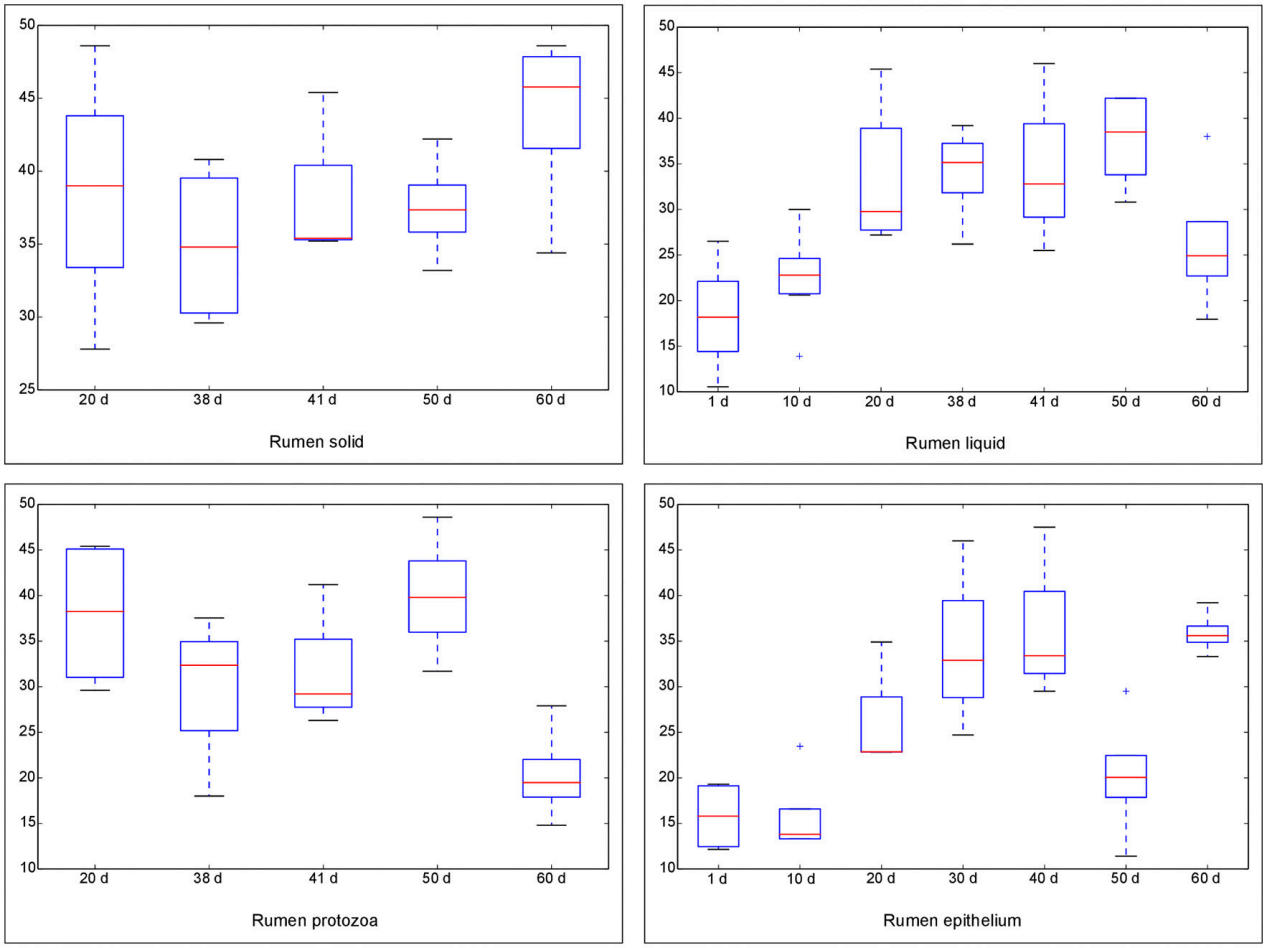
**Comparison of Chao 1 index of bacterial communities within different ages for each fraction**. The horizontal lines in each box indicate the median values, and the 75 and 25th quartile values are respectively represented by the top and bottom sides of each box.

**Figure 2:**
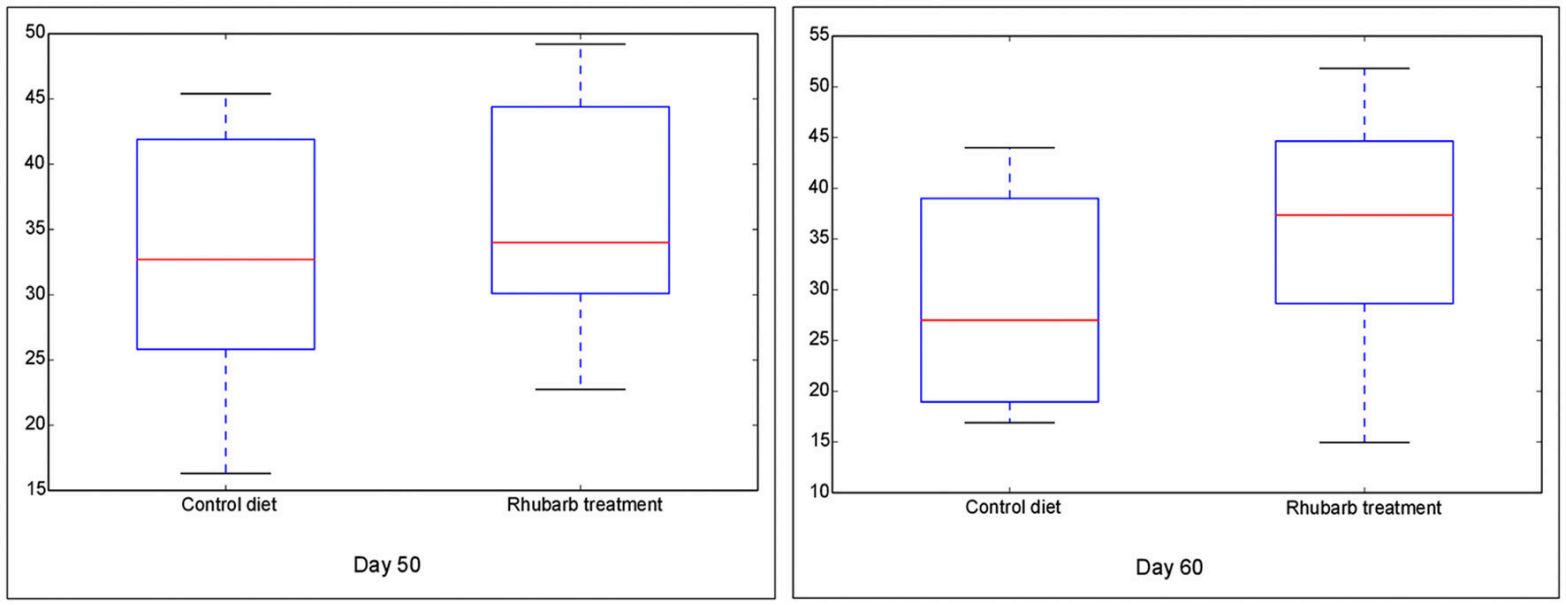
**Comparison of Chao 1 index of bacterial communities between control diet and rhubarb treatment on 50 and 60 d**. The horizontal lines in each box indicate the median values, and the 75 and 25th quartile values are respectively represented by the top and bottom sides of each box.

The Chao 1 index of Alpha diversity was used to measure and compare the bacterial diversity within different days in each fraction (Figure 1). An age-dependent rise of Chao 1 was positively observed in the fractions of RL and RE, but no similar pattern was shown in RS and RP. Furthermore, the comparison of Chao 1 within four fractions on different days revealed that at 50 d the Chao 1 richness in RE was the lowest compared to other three fractions, and at 60 d the richness in RP was less than the other three fractions (Figure S7). There was no difference in the Chao 1 indices between the control diet and rhubarb treatment on 50 or 60 d (Figure 2).

**Figure 3:**
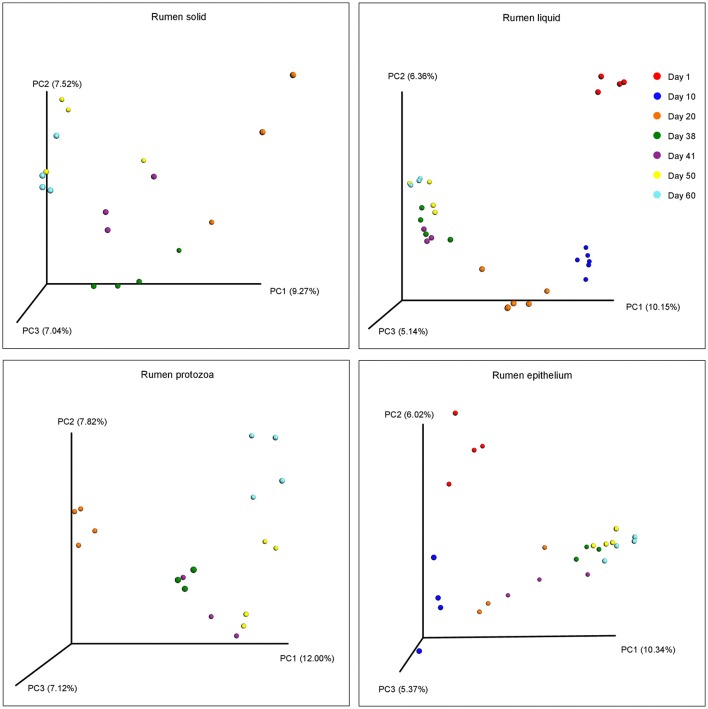
**Principal coordinate analysis (PCoA) of bacterial community structure using unweighted Unifrac matrix within different ages for each fraction**.

**Figure 4:**
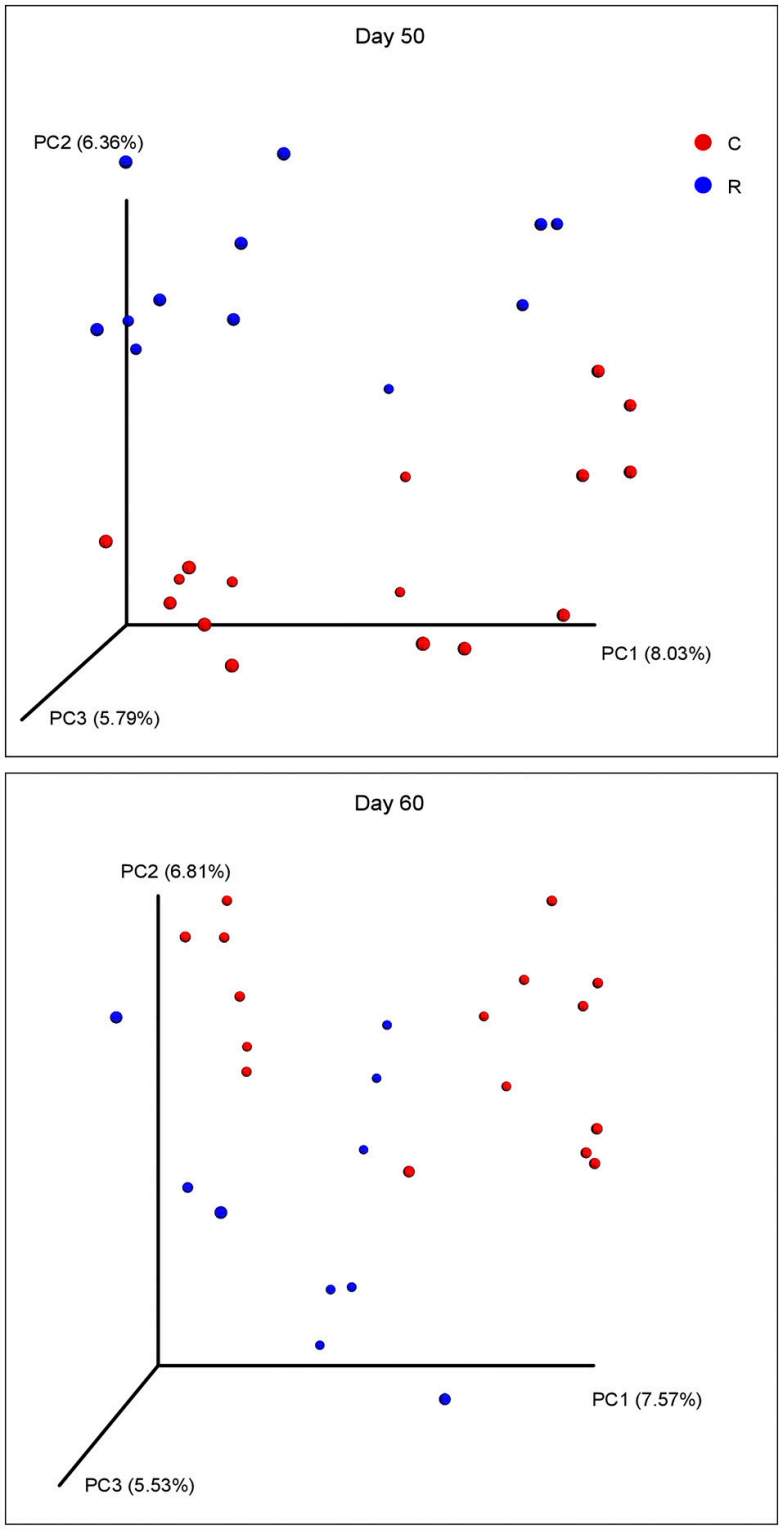
**Principal coordinate analysis (PCoA) of bacterial community structure using unweighted Unifrac matrix between two treatments on 50 and 60 d**.

The beta diversities of bacteria communities within different ages for each fraction were calculated and visualized through three dimensional PCoA analysis using the unweighted UniFrac distances (Figure 3). The samples in all the four fractions clustered based on different age groups. Moreover, the bacterial communities on 38 d and 41 d were separate in RS and RL, while no distinction between these two age groups was found in RP or RE. Difference between the bacterial communities in RE and RL was observed on 1 d and 10 d (Figure S8), and the community in RE was always different from the communities in other three fractions which clustered together. On 50 d, a clear distinction between the bacterial communities in the control diet treatment and the rhubarb treatment was noted (Figure 4). However, there was no similar treatment-dependent clustering pattern between two treatments on 60 d.

### Relative abundance of bacteria in four fractions during rumen development and with rhubarb treatment

After the arcsine transformation and subsequent statistical analysis, all the data at phylum level was converted back into the original percent relative abundance and is presented in Table 1. Result of the statistical analysis indicated that the relative abundances of all the main bacterial phyla, except Fibrobacteres, were significantly (*P* < 0.01) affected by the fraction of samples, and a significant (*P* < 0.05) interaction between fraction and age was observed for the relative abundances of the phyla *Bacteroidetes, Spirochaetae*, and Synergistetes. For the phylum *Proteobacteria*, its abundance in RE was always the greatest compared to other three fractions, and different types of decline in abundances over time were, respectively, noted in RS (cubic, *P* < 0.05), RL (cubic, *P* < 0.05), RP (linear, *P* < 0.01), and RE (quadratic, *P* < 0.01). Further, the abundances of *Proteobacteria* in four fractions on 41 d were significantly (*P* < 0.05) or numerically higher than those on 38 d. The relative abundance of *Firmicutes* in RE was generally less than those in the other three fractions, cubic (*P* < 0.05), linear (*P* < 0.01), and quadratic (*P* < 0.01) increments with age were shown in RS, RL, and RE, respectively. However, significant (*P* < 0.05) or numerical decreases of abundances in four fractions from 38 to 41 d were also observed. Age exerted quadratic effects on the abundances of *Bacteroidetes* in RL (*P* < 0.01), RP (*P* < 0.05), and RE (*P* < 0.05). The relative abundances of Fibrobacteres in RP (*P* < 0.05) and RE (*P* < 0.01) both linearly decreased as the age increased. For the phylum *Spirochaetae*, its abundance in RS was always the highest from 20 to 60 d, and different increases of abundances were found in RL (quadratic, *P* < 0.05), RP (cubic, *P* < 0.01), and RE (linear, *P* < 0.01), respectively. The abundances of *Actinobacteria* in RS and RP on 41 d were significantly (*P* < 0.05) improved compared with those on 38 d, and the abundances in RP and RE both increased quadratically (*P* < 0.05) with age, while a cubic (*P* < 0.05) effect of age was observed in RL. An increasing quadratic (*P* < 0.01) effect of age on the relative abundance of Synergistetes was presented in RS, RP, and RE, while the abundance of Synergistetes in RL rose cubically (*P* < 0.01) with age.

**Table 1:** **Change of relative abundance (%) of bacterial phyla in four fractions during rumen development**.

**Fraction**	**Age (d)**							**SEM^1^**	**Significance (*P*<)**^2^		
	**1**	**10**	**20**	**38**	**41**	**50**	**60**		**Fraction**	**Age**	**Fraction × Age**
***PROTEOBACTERIA***											
RS	–^3^	–	8.49^c^	1.33^b^	10.79^b^	11.67^b^	3.71^b^	0.810	<0.01	C (<0.05)	NS
RL	70.25^b^	47.13^b^	17.38^bc^	2.17^b^	19.62^ab^	13.83^b^	4.12^b^			C (<0.05)	
RP	–	–	25.80^ab^	1.95^b^	19.86^ab^	10.33^b^	1.20^b^			L (<0.01)	
RE	89.05^a^	78.79^a^	47.14^a^	18.97^a^	37.33^a^	56.37^a^	26.13^a^			Q (<0.01)	
***FIRMICUTES***											
RS	–	–	47.07	66.73^a^	35.51	41.85^a^	55.70^b^	0.574	<0.01	C (<0.05)	NS
RL	12.20	20.86^a^	38.61	55.88^ab^	44.47	41.06^a^	61.69^ab^			L (<0.01)	
RP	–	–	46.48	63.33^a^	35.53	46.87^a^	73.44^a^			NS	
RE	6.65	8.59^b^	36.97	43.02^b^	39.61	23.55^b^	44.86^b^			Q (<0.01)	
***BACTEROIDETES***											
RS	–	–	27.37^ab^	24.86	28.73^a^	30.66^a^	18.92^a^	0.478	<0.01	NS	<0.05
RL	14.28^a^	29.44^a^	35.59^a^	35.86	27.61^a^	29.96^a^	10.69^ab^			Q (<0.01)	
RP	–	–	19.34^bc^	26.90	19.59^ab^	25.52^a^	6.20^b^			Q (<0.05)	
RE	2.31^b^	6.27^b^	11.76^c^	26.82	10.31^b^	11.88^b^	15.55^ab^			Q (<0.05)	
**FIBROBACTERES**											
RS	–	–	5.09	0.02	1.55	1.03	0.11	0.176	NS	NS	NS
RL	0.08^b^	0.08	1.28	0.11	0.79	1.06	0.07			NS	
RP	–	–	1.55	0.72	0.26	0.51	0.12			L (<0.05)	
RE	0.82^a^	0.31	1.54	0.15	0.62	0.17	0.13			L (<0.01)	
***SPIROCHAETAE***											
RS	–	–	7.02^a^	1.69^a^	5.48^a^	4.53^a^	2.97^a^	0.070	<0.01	NS	<0.05
RL	0.02	0.31	1.52^b^	0.71^ab^	1.36^b^	2.96^a^	1.05^b^			Q (<0.05)	
RP	–	–	0.38^c^	0.52^b^	0.81^b^	1.14^b^	0.16^c^			C (<0.01)	
RE	0.28	0.27	0.84^bc^	0.60^ab^	1.13^b^	1.23^b^	2.88^a^			L (<0.01)	
***ACTINOBACTERIA***											
RS	–	–	1.73	3.32	15.19^a^	2.52	4.49	0.356	<0.01	NS	NS
RL	2.49	0.78	0.62	2.90	1.63^b^	0.94	0.43			C (<0.05)	
RP	–	–	2.21	5.71	22.40^a^	3.87	1.10			Q (<0.05)	
RE	0.44	2.96	1.24	8.68	9.36^a^	2.28	3.49			Q (<0.05)	
**SYNERGISTETES**											
RS	–	–	1.54	1.01	0.66	6.90	13.62^ab^	0.279	<0.01	Q (<0.01)	<0.05
RL	0.02	0.24	1.81	1.95	1.09	9.17	21.10^a^			C (<0.01)	
RP	–	–	2.89	0.51	0.55	10.94	17.52^a^			Q (<0.01)	
RE	0.31	0.12	0.21	1.26	0.56	4.28	6.63^b^			Q (<0.01)	

a, b, c *Means within a column for fractions that do not have a common superscript differ (P <0.05)*.

1 SEM for fraction × age;

2 NS, not significant (P > 0.05); L, Linear effect of age; Q, Quadratic effect of age; C, Cubic effect of age;

3 *No data due to the absence of samples*.

The supplementation of rhubarb root powder significantly (*P* < 0.05) affected the relative abundance of *Proteobacteria* throughout four fractions, and a significant (*P* < 0.05) interaction between diet and age was revealed on the relative abundances of *Proteobacteria* and *Firmicutes* (Table 2). In the rhubarb treatment at 50 d, the abundance of *Proteobacteria* in RE was significantly (*P* < 0.05) reduced compared to that of the control diet, while the abundance of *Firmicutes* in RE and the abundance of *Bacteroidetes* in RL were significantly (*P* < 0.05) higher than the control. Moreover, the supplementation of rhubarb significantly (*P* < 0.05) decreased the abundance of *Spirochaetae* in RE when compared with the control on 60 d. It is also noted that the abundances of Synergistetes in all the four fractions of the rhubarb treatment on 50 d were numerically less than those of the control diet.

**Table 2:** **Comparison of relative abundance (%) of bacterial phyla between control diet and rhubarb treatment**.

**Fraction**	**Diet**	**Age (d)**		**SEM^1^**	**Significance (*P*<)**^2^		
		**50**	**60**		**Diet**	**Age**	**Diet × Age**
***PROTEOBACTERIA***							
RS	C^3^	11.67	3.71	0.178	<0.05	NS	<0.05
	R^4^	4.59	2.58				
RL	C	13.83	4.12			NS	
	R	13.55	6.90				
RP	C	10.33	1.20			<0.05	
	R	3.72	2.10				
RE	C	56.37^a^	26.13			NS	
	R	22.49^b^	24.24				
***FIRMICUTES***							
RS	C	41.85	55.70	0.071	NS	<0.05	<0.05
	R	51.36	61.40				
RL	C	41.06	61.69			<0.01	
	R	29.88	49.24				
RP	C	46.87	73.44			<0.05	
	R	57.84	61.85				
RE	C	23.55^b^	44.86			NS	
	R	50.01^a^	52.46				
***BACTEROIDETES***							
RS	C	30.66	18.92	0.103	NS	<0.05	NS
	R	30.01	20.37				
RL	C	29.96^b^	10.69			<0.01	
	R	47.19^a^	17.71				
RP	C	25.52	6.20			<0.01	
	R	26.41	9.66				
RE	C	11.88	15.55			NS	
	R	17.49	11.08				
**FIBROBACTERES**							
RS	C	1.03	0.11	0.024	NS	NS	NS
	R	0.06	0.06				
RL	C	1.06	0.07			NS	
	R	0.45	0.92				
RP	C	0.51	0.12			NS	
	R	0.05	0.03				
RE	C	0.17	0.13			NS	
	R	0.09	0.18				
***SPIROCHAETAE***							
RS	C	4.53	2.97	0.010	NS	<0.05	NS
	R	4.21	3.03				
RL	C	2.96	1.05			<0.05	
	R	2.50	1.15				
RP	C	1.14	0.16			<0.01	
	R	0.95	0.23				
RE	C	1.23	2.88^a^			NS	
	R	1.36	0.91^b^				
***ACTINOBACTERIA***							
RS	C	2.52	4.49	0.032	NS	NS	NS
	R	2.89	2.57				
RL	C	0.94	0.43			NS	
	R	0.82	0.41				
RP	C	3.87	1.10			<0.05	
	R	2.36	1.10				
RE	C	2.28	3.49			NS	
	R	4.47	2.53				
**SYNERGISTETES**							
RS	C	6.90	13.62	0.168	NS	NS	NS
	R	4.97	9.00				
RL	C	9.17	21.10			<0.01	
	R	4.17	18.75				
RP	C	10.94	17.52			NS	
	R	6.94	22.64				
RE	C	4.28	6.63			NS	
	R	2.97	7.62				

a, b *Means within a column for diets that do not have a common superscript differ (P <0.05)*.

1 SEM for diet × age;

2 NS, not significant (P > 0.05);

3 C, Control diet;

4 *R, Rhubarb treatment*.

Data at genus level was converted back into the original percent relative abundance after the statistical analysis and is displayed in Table 3. Except the genera *Arcobacter, Bibersteinia, Lactobacillus, Ruminiclostridium, Streptococcus*, and *Fibrobacter*, the distributions of all the remaining 24 genera with relative abundances >0.5% were significantly (*P* < 0.05 or 0.01) influenced by the sample fraction. Furthermore, significant (*P* < 0.05 or 0.01) interactions between fraction and age were, respectively, found on the relative proportions of the genera *Actinobacillus, Alysiella, Arcobacter, Bibersteinia, Mannheimia, Moraxella, Suttonella, Butyrivibrio, Christensenellaceae R-7 group, Quinella, Ruminococcaceae NK4A214 group, Ruminococcus, Streptococcus, Bacteroides, Porphyromonas, Prevotella, Prevotellaceae UCG-001, Fretibacterium*, and *Treponema*. In accordance with the fact that *Proteobacteria* abundance decreased significantly (*P* < 0.05) with age, declines to different extents in the relative abundances within four fractions of the genera *Actinobacillus, Alysiella, Arcobacter, Bibersteinia, Mannheimia*, and *Moraxella* were observed, respectively. Nevertheless, the proportions of the *Ruminobacter* spp. in four fractions significantly (*P* < 0.05) or numerically increased, and the abundances of the *Campylobacter* (cubic, *P* < 0.05) and *Suttonella* (quadratic, *P* < 0.05) phylotypes in RE rose as the age increased. It is also observed that the fraction of RE accommodated the greatest proportion of *Campylobacter* spp. compared to other three fractions from 20 to 60 d, and the abundance of *Suttonella* was significantly (*P* < 0.05) or numerically higher than those in the other three fractions for most of the days. Within the phylum *Firmicutes*, the relative abundances of the *Lactobacillus* spp. and *Streptococcus* spp. in four fractions generally underwent decreases in different patterns. On the contrary, age had different increasing effects on the relative proportions of the genera *Acetitomaculum, Blautia, Lachnospiraceae NK3A20 group, Quinella, Ruminiclostridium, Ruminococcaceae NK4A214 group*, and *Ruminococcus* in four fractions. When compared to 38 d, the abundances of the genera *Lachnospiraceae NK3A20 group* and *Quinella* in four fractions on 41 d were significantly (*P* < 0.05) reduced, while the proportion of *Ruminiclostridium* spp. was significantly (*P* < 0.05) enhanced. For the *Christensenellaceae R-7 group* phylotypes, a cubic (*P* < 0.01) effect of age on its abundances in RL and RE was presented, while its proportions in RS (*P* < 0.05) and RP (*P* < 0.01) reduced quadratically with age. The abundance of *Succiniclasticum* spp. in RS declined linearly (*P* < 0.01) with age, while age exerted quadratic (*P* < 0.01) and linear (*P* < 0.01) influences on the proportions in RL and RE, respectively. Moreover, RE harbored the most *Butyrivibrio* spp. but basically the least *Quinella* spp. compared with the other three fractions, while the abundance of the *Blautia* phylotypes in RS was the highest from 38 to 60 d. In the *Bacteroidetes* phylum, the relative proportions of the genera *Prevotella* and *Prevotellaceae UCG-001* in four fractions increased to different extents with age, while the decrease in the abundance of *Porphyromonas* spp. was noted. The proportions of *Prevotella* in four fractions and *Prevotellaceae UCG-001* in RL and RE on 41 d were significantly (*P* < 0.05) or numerically lower than those on 38 d. For the genus *Bacteroides*, its abundances in RS (*P* < 0.05) and RP (*P* < 0.01) underwent linear decline with age, while a cubic (*P* < 0.01) effect of age on the abundances in RL and RE was also noticed. For the most of the days during rumen development, the abundance of the *Prevotellaceae UCG-001* phylotypes in RE was significantly (*P* < 0.05) or numerically higher, while the ratio of *Prevotella* in RE was significantly (*P* < 0.05) or numerically fewer than those in the other three fractions. In the rest 4 minor phyla, the abundances of *Fibrobacter* spp. in RP (*P* < 0.05) and RE (*P* < 0.01) decreased linearly with age, while different increasing effects of age on the proportions of *Treponema* in RL, RP, and RE were, respectively, observed. Different manners of increases in the relative abundances of the *Atopobium* spp. in four fractions were noted, and the abundances in RS, RP, and RE reached their peak on 41 d. In contrast, the proportions of *Fretibacterium* in all the four fractions quadratically (*P* < 0.05 or 0.01) increased with age, achieving their maxima on 60 d.

**Table 3:** **Change of relative abundance (%) of bacterial genera in four fractions during rumen development**.

**Phylum**	**Genus**	**Fraction**	**Age (d)**							**SEM^1^**	**Significance (*P*<)**^2^		
			**1**	**10**	**20**	**38**	**41**	**50**	**60**		**Fraction**	**Age**	**Fraction × Age**
*Proteobacteria*	*Actinobacillus*	RS	– ^3^	–	0.11^b^	0.00	0.01	0.00	0.01	0.012	<0.01	Q (<0.05)	<0.05
		RL	1.34^b^	0.10	0.13^b^	0.01	0.01	0.00	0.00			C (<0.01)	
		RP	–	–	0.38^a^	0.04	0.01	0.01	0.01			Q (<0.01)	
		RE	2.61^a^	0.12	0.04^b^	0.00	0.02	0.00	0.00			C (<0.01)	
	*Alysiella*	RS	–	–	0.00^c^	0.00	0.00^b^	0.00^b^	0.00	0.280	<0.01	NS	<0.05
		RL	1.68^b^	0.10	0.07^b^	0.00	0.01^b^	0.00^b^	0.00			Q (<0.05)	
		RP	–	–	0.17^b^	0.03	0.01^b^	0.00^b^	0.00			L (<0.01)	
		RE	9.7^a^	0.22	0.60^a^	0.01	0.10^a^	0.08^a^	0.01			NS	
	*Arcobacter*	RS	–	–	0.01	0.00	0.00	0.01	0.01	0.165	NS	NS	<0.01
		RL	0.01	9.05^a^	0.01	0.01	0.00	0.01	0.00			C (<0.05)	
		RP	–	–	0.01	0.00	0.02	0.00	0.01			NS	
		RE	0.01	0.34^b^	0.06	0.00	0.01	0.01	0.02			C (<0.05)	
	*Bibersteinia*	RS	–	–	1.40^c^	0.01	0.08	0.00	0.02	0.409	NS	NS	<0.05
		RL	10.7^b^	0.35	1.68^b^	0.01	0.07	0.00	0.00			Q (<0.05)	
		RP	–	–	2.85^a^	0.09	0.04	0.01	0.01			Q (<0.01)	
		RE	27.7^a^	1.03	0.25^c^	0.02	0.09	0.02	0.00			C (<0.05)	
	*Campylobacter*	RS	–	–	0.15^b^	0.01^b^	0.04^b^	0.01^b^	0.01^b^	0.444	<0.01	L (<0.01)	NS
		RL	0.01	14.41	1.72^b^	0.06^b^	0.02^b^	0.02^b^	0.02^b^			C (<0.01)	
		RP	–	–	1.36^b^	0.13^b^	0.10^b^	0.02^b^	0.02^b^			Q (<0.01)	
		RE	0.06	10.96	8.48^a^	4.48^a^	5.42^a^	4.38^a^	6.47^a^			C (<0.05)	
	*Mannheimia*	RS	–	–	0.21^b^	0.00^b^	0.03	0.00	0.00	0.288	<0.01	NS	<0.05
		RL	27.11^a^	0.29	0.31^b^	0.01^b^	0.03	0.01	0.01			C (<0.01)	
		RP	–	–	0.63^a^	0.05^a^	0.01	0.01	0.01			Q (<0.01)	
		RE	20.07^b^	0.09	0.12^b^	0.03^ab^	0.04	0.01	0.01			C (<0.05)	
	*Moraxella*	RS	–	–	0.13^b^	0.01	0.00^b^	0.00	0.00	0.322	<0.05	Q (<0.05)	<0.01
		RL	9.67	0.53	0.20^b^	0.01	0.02^ab^	0.01	0.00			Q (<0.05)	
		RP	–	–	0.49^a^	0.03	0.01^ab^	0.00	0.00			L (<0.01)	
		RE	6.04	0.09	0.06^b^	0.00	0.06^a^	0.00	0.02			L (<0.01)	
	*Ruminobacter*	RS	–	–	1.18	0.33	5.60^ab^	6.74	1.25	0.561	<0.01	C (<0.05)	NS
		RL	0.02	0.03	4.27	0.22	15.71^a^	7.61	1.70			L (<0.01)	
		RP	–	–	4.57	0.09	16.80^a^	7.95	0.43			NS	
		RE	0.08	0.02	0.85	0.34	0.38^b^	1.26	0.33			L (<0.05)	
	*Suttonella*	RS	–	–	0.01	0.07^b^	0.00	0.14^b^	0.03^b^	0.188	<0.01	NS	<0.01
		RL	0.00	0.01	0.01	0.17^ab^	0.00	0.16^b^	0.06^b^			L (<0.01)	
		RP	–	–	0.02	0.00^b^	0.00	0.23^b^	0.07^b^			C (<0.01)	
		RE	0.01	0.02	0.15	3.38^a^	0.02	36.33^a^	12.02^a^			Q (<0.05)	
*Firmicutes*	*Acetitomaculum*	RS	–	–	1.26	3.31^a^	2.79	2.05^a^	1.35^a^	0.075	<0.01	NS	NS
		RL	0.01	0.01	0.57	1.30^b^	1.98	0.42^b^	0.18^b^			C (<0.05)	
		RP	–	–	0.28	2.67^ab^	2.81	1.81^a^	0.61^ab^			Q (<0.01)	
		RE	0.09	0.05	0.75	2.10^ab^	1.78	1.24^ab^	0.85^ab^			L (<0.01)	
	*Blautia*	RS	–	–	0.57	2.39^a^	2.05^a^	1.43^a^	0.85^a^	0.042	<0.01	Q (<0.05)	NS
		RL	0.03	0.08	0.22	0.97^bc^	0.59^b^	0.47^bc^	0.14^b^			C (<0.05)	
		RP	–	–	0.33	1.61^ab^	1.32^ab^	1.30^ab^	0.22^ab^			Q (<0.01)	
		RE	0.03	0.03	0.09	0.32^c^	0.53^b^	0.30^c^	0.35^ab^			L (<0.01)	
	*Butyrivibrio*	RS	–	–	0.72^b^	0.46^b^	0.17^b^	0.16^b^	0.20^b^	0.161	<0.01	L (<0.01)	<0.01
		RL	0.01	0.01	0.36^b^	0.42^b^	0.14^b^	0.14^b^	0.10^b^			Q (<0.01)	
		RP	–	–	0.49^b^	0.16^b^	0.07^b^	0.03^b^	0.03^b^			L (<0.01)	
		RE	0.04	0.06	6.04^a^	10.61^a^	13.42^a^	2.19^a^	6.33^a^			Q (<0.01)	
	*Christensenellaceae R-7 group*	RS	–	–	6.40^bc^	3.71^a^	2.10	1.52	4.39^a^	0.132	<0.05	Q (<0.05)	<0.01
		RL	0.05	1.68	4.53^c^	3.47^a^	2.25	0.40	0.73^b^			C (<0.01)	
		RP	–	–	23.76^a^	1.85^ab^	1.47	0.73	0.61^b^			Q (<0.01)	
		RE	0.27	2.38	10.17^b^	0.73^b^	2.09	0.43	1.19^b^			C (<0.01)	
	*Lachnospiraceae NK3A20 group*	RS	–	–	3.66	21.49^a^	6.03	6.75	8.33^a^	0.369	<0.05	C (<0.05)	NS
		RL	0.06	0.06	2.59	11.79^b^	2.94	3.61	1.29^b^			Q (<0.01)	
		RP	–	–	0.44	17.93^ab^	5.75	8.19	3.20^ab^			Q (<0.01)	
		RE	0.34	0.15	3.15	9.29^b^	3.83	3.11	5.91^ab^			L (<0.01)	
	*Lactobacillus*	RS	–	–	0.03	0.00	0.01	0.00	0.01	0.014	NS	NS	NS
		RL	0.50	0.07	0.01	0.01	0.01	0.00	0.01			L (<0.05)	
		RP	–	–	0.01	0.00	0.00	0.00	0.02			Q (<0.01)	
		RE	0.33	0.02	0.01	0.00	0.01	0.00	0.00			Q (<0.05)	
	*Quinella*	RS	–	–	0.02	10.18^ab^	1.98	4.41^ab^	10.59^b^	0.825	<0.01	L (<0.01)	<0.01
		RL	0.02	0.02	0.03	16.88^a^	6.02	11.24^ab^	36.00^a^			Q (<0.05)	
		RP	–	–	0.06	22.14^a^	2.40	16.07^a^	49.58^a^			L (<0.01)	
		RE	0.45	0.08	0.13	1.73^b^	0.93	1.67^b^	6.01^b^			Q (<0.01)	
	*Ruminiclostridium*	RS	–	–	7.94^a^	0.64	2.42	0.56	1.05	0.244	NS	L (<0.05)	NS
		RL	0.02	1.79	4.06^ab^	0.44	5.66	0.23	0.43			Q (<0.05)	
		RP	–	–	0.74^b^	0.20	3.35	0.32	0.17			NS	
		RE	0.14	0.91	5.05^ab^	0.10	0.92	0.23	0.35			NS	
	*Ruminococcaceae NK4A214 group*	RS	–	–	4.33^b^	3.07^a^	2.73	2.54	4.10	0.094	<0.01	NS	<0.01
		RL	0.03	5.01^a^	12.34^a^	1.73^ab^	3.83	1.62	2.42			C (<0.01)	
		RP	–	–	1.05^c^	3.41^a^	3.28	2.09	2.67			NS	
		RE	0.46	0.55^b^	2.33^bc^	1.00^b^	1.68	0.95	1.95			L (<0.05)	
	*Ruminococcus*	RS	–	–	5.24	4.17^ab^	3.40^ab^	6.95^a^	9.71^a^	0.178	<0.01	NS	<0.05
		RL	0.05	0.43	1.84	6.50^a^	7.29^a^	4.83^ab^	7.86^a^			Q (<0.05)	
		RP	–	–	3.14	1.71^b^	4.91^ab^	4.75^ab^	2.54^b^			NS	
		RE	0.44	0.17	2.87	2.07^ab^	2.16^b^	1.51^b^	5.49^ab^			L (<0.01)	
	*Selenomonas*	RS	–	–	0.31	0.53	0.36	1.51^ab^	0.55	0.050	<0.01	NS	NS
		RL	0.00	0.00	0.14	0.25	0.37	2.54^a^	0.56			L (<0.01)	
		RP	–	–	0.01	0.07	0.11	0.94^bc^	0.09			NS	
		RE	0.01	0.01	0.10	0.53	0.12	0.35^c^	0.24			L (<0.01)	
	*Streptococcus*	RS	–	–	0.19^bc^	0.01	0.08^b^	0.02	0.01	0.045	NS	Q (<0.05)	<0.01
		RL	6.52^a^	0.45	0.21^b^	0.01	0.05^b^	0.01	0.00			C (<0.01)	
		RP	–	–	0.98^a^	0.03	0.09^b^	0.01	0.00			Q (<0.05)	
		RE	1.69^b^	0.20	0.04^c^	0.00	0.46^a^	0.01	0.01			C (<0.01)	
	*Succiniclasticum*	RS	–	–	2.18^a^	1.02	1.05^a^	0.93	0.47	0.039	<0.01	L (<0.01)	NS
		RL	0.01	0.18	1.71^ab^	0.35	0.44^ab^	0.72	0.19			Q (<0.01)	
		RP	–	–	0.72^bc^	0.98	0.44^ab^	1.30	0.15			NS	
		RE	0.05	0.02	0.47^c^	0.42	0.21^b^	0.59	0.33			L (<0.01)	
*Bacteroidetes*	*Bacteroides*	RS	–	–	3.43^ab^	1.23^ab^	0.59	0.93	0.85	0.110	<0.01	L (<0.05)	<0.01
		RL	0.02	10.44^a^	6.60^a^	1.31^ab^	0.58	0.88	0.30			C (<0.01)	
		RP	–	–	7.20^a^	3.21^a^	0.57	1.84	0.44			L (<0.01)	
		RE	0.07	1.03^b^	0.66^b^	0.33^b^	0.23	0.37	0.56			C (<0.01)	
	*Porphyromonas*	RS	–	–	0.19^b^	0.20^b^	0.02	0.01	0.01	0.153	<0.01	NS	<0.01
		RL	3.47^a^	5.52^a^	0.65^a^	0.18^b^	0.03	0.00	0.00			L (<0.01)	
		RP	–	–	0.67^a^	0.49^a^	0.03	0.01	0.01			L (<0.01)	
		RE	0.15^b^	1.20^b^	0.26^b^	0.18^b^	0.13	0.00	0.01			L (<0.05)	
	*Prevotella*	RS	–	–	6.59	14.99^ab^	12.97^a^	20.36^a^	10.8^a^	0.309	<0.01	Q (<0.05)	<0.01
		RL	0.08	1.96	4.99	21.22^a^	15.64^a^	22.15^a^	6.92^ab^			C (<0.01)	
		RP	–	–	1.27	13.22^ab^	7.46^ab^	15.95^a^	3.34^b^			Q (<0.01)	
		RE	0.39	0.39	4.08	6.46^b^	2.49^b^	6.80^b^	6.15^ab^			L (<0.01)	
	*Prevotellaceae UCG-001*	RS	–	–	1.09	1.25^b^	1.83^ab^	0.94	0.81^b^	0.095	<0.01	NS	<0.01
		RL	0.02	0.22	0.69	2.04^b^	1.07^b^	0.72	0.85^b^			Q (<0.01)	
		RP	–	–	0.56	1.15^b^	2.33^ab^	0.70	0.28^b^			Q (<0.01)	
		RE	0.09	0.36	0.92	16.33^a^	3.2^a^	1.6	5.45^a^			Q (<0.01)	
Fibrobacteres	*Fibrobacter*	RS	–	–	5.09	0.02	1.55	1.03	0.11	0.176	NS	NS	NS
		RL	0.08^b^	0.08	1.28	0.11	0.79	1.06	0.07			NS	
		RP	–	–	1.55	0.72	0.26	0.51	0.12			L (<0.05)	
		RE	0.82^a^	0.31	1.54	0.15	0.62	0.17	0.13			L (<0.01)	
*Spirochaetae*	*Treponema*	RS	–	–	6.97^a^	1.68^a^	5.42^a^	4.50^a^	2.96^a^	0.071	<0.01	NS	<0.05
		RL	0.01	0.18	1.40^b^	0.69^ab^	1.31^b^	2.94^ab^	1.03^b^			Q (<0.05)	
		RP	–	–	0.35^c^	0.52^b^	0.79^b^	1.12^c^	0.15^c^			C (<0.05)	
		RE	0.26	0.05	0.78^bc^	0.58^ab^	1.12^b^	1.23^bc^	2.87^a^			L (<0.01)	
*Actinobacteria*	*Atopobium*	RS	–	–	0.24	2.59	12.73^a^	1.94	3.73	0.300	<0.01	NS	NS
		RL	0.01	0.01	0.14	2.38	1.26^b^	0.72	0.30			C (<0.05)	
		RP	–	–	0.25	4.52	19.29^a^	3.12	0.85			Q (<0.05)	
		RE	0.11	0.03	0.38	1.57	2.54^b^	0.83	1.55			L (<0.01)	
Synergistetes	*Fretibacterium*	RS	–	–	0.91	0.92	0.37	6.42^a^	13.28^a^	0.268	<0.01	Q (<0.01)	<0.01
		RL	0.01	0.08	0.16	1.92	0.83	8.90^a^	20.99^a^			Q (<0.01)	
		RP	–	–	0.38	0.33	0.20	10.53^a^	17.35^a^			Q (<0.05)	
		RE	0.28	0.09	0.06	1.21	0.43	1.61^b^	6.41^b^			Q (<0.01)	

a, b, c *Means within a column for fractions that do not have a common superscript differ (P <0.05)*.

1 SEM for fraction × age;

2 NS, not significant (P > 0.05), L, Linear effect of age, Q, Quadratic effect of age, C, Cubic effect of age;

3 *No data due to the absence of samples*.

On the genus level, the addition of rhubarb significantly (*P* < 0.05 or 0.01) influenced the relative proportions throughout fractions of the genera *Actinobacillus, Suttonella, Butyrivibrio, Christensenellaceae R-7 group, Lachnospiraceae NK3A20 group, Quinella*, and *Ruminococcaceae NK4A214 group* (Table 4), and significant interaction between diet and age was observed on the abundances of *Suttonella* (*P* < 0.05), *Lachnospiraceae NK3A20 group* (*P* < 0.05), *Ruminiclostridium* (*P* < 0.01), and *Selenomonas* (*P* < 0.05). On 50 d in the rhubarb treatment, the proportions of the *Campylobacter* spp. and *Suttonella* spp. in RE, and *Selenomonas* spp. in RL were significantly (*P* < 0.05) reduced compared with those of the control diet, while the relative abundances of *Butyrivibrio* spp. and *Prevotellaceae UCG-001* in RE, *Christensenellaceae R-7 group* in RS and RE, Lachnospiraceae NK3A20 group in RS, RP, and RE, and *Ruminococcaceae NK4A214 group* in RS and RP were greater (*P* < 0.05) than those of the control. On 60 d, the relative proportions of *Actinobacillus, Christensenellaceae R-7 group*, and *Ruminococcaceae NK4A214 group* in RL, and *Bibersteinia* and *Butyrivibrio* in RE were significantly (*P* < 0.05) improved by the supplementation of rhubarb, while the abundances of *Quinella* in RL and *Treponema* in RE of the rhubarb treatment were lower (*P* < 0.05) than those of the control diet. Moreover, the addition of rhubarb significantly (*P* < 0.05) enhanced the abundance of *Ruminiclostridium* spp. in RL on 50 d, however a reverse effect on its proportions in RS and RE was observed as well.

**Table 4:** **Comparison of relative abundance (%) of bacterial genera between control diet and rhubarb treatment**.

**Phylum**	**Genus**	**Fraction**	**Age (d)**				**SEM^1^**	**Significance (P<)^2^**		
			**50**		**60**			**Diet**	**Age**	**Diet × Age**
			**C^3^**	**R^4^**	**C**	**R**				
*Proteobacteria*	*Actinobacillus*	RS	0.00	0.01	0.01	0.00	0.0006	<0.05	NS	NS
		RL	0.00	0.00	0.00^b^	0.13^a^			NS	
		RP	0.01	0.00	0.01	0.01			NS	
		RE	0.00	0.01	0.00	0.02			NS	
	*Alysiella*	RS	0.00	0.00	0.00	0.00	0.0005	NS	NS	NS
		RL	0.00	0.00	0.00	0.00			NS	
		RP	0.00	0.00	0.00	0.00			NS	
		RE	0.08	0.03	0.01	0.01			NS	
	*Arcobacter*	RS	0.01	0.00	0.01	0.00	0.0006	NS	NS	NS
		RL	0.01	0.01	0.00	0.00			NS	
		RP	0.00	0.00	0.01	0.00			NS	
		RE	0.01	0.00	0.02	0.04			NS	
	*Bibersteinia*	RS	0.00	0.00	0.02	0.01	0.0007	NS	<0.05	NS
		RL	0.00	0.01	0.00	0.02			NS	
		RP	0.01	0.00	0.01	0.01			<0.05	
		RE	0.02	0.02	0.00^b^	0.03^a^			NS	
	*Campylobacter*	RS	0.01	0.01	0.01	0.01	0.0256	NS	NS	NS
		RL	0.02	0.13	0.02	0.28			NS	
		RP	0.02	0.02	0.02	0.02			NS	
		RE	4.38^a^	0.62^b^	6.47	4.56			NS	
	*Mannheimia*	RS	0.00	0.01	0.00	0.01	0.0006	NS	NS	NS
		RL	0.01	0.02	0.01	0.04			NS	
		RP	0.01	0.01	0.01	0.00			NS	
		RE	0.01	0.01	0.01	0.00			NS	
	*Moraxella*	RS	0.00	0.00	0.00	0.00	0.0003	NS	NS	NS
		RL	0.01	0.01	0.00	0.00			NS	
		RP	0.00	0.00	0.00	0.01			NS	
		RE	0.00	0.00	0.02	0.00			NS	
	*Ruminobacter*	RS	6.74	3.02	1.25	1.35	0.0849	NS	NS	NS
		RL	7.61	7.32	1.70	1.55			NS	
		RP	7.95	2.10	0.43	0.79			<0.05	
		RE	1.26	1.08	0.33	0.41			NS	
	*Suttonella*	RS	0.14	0.07	0.03	0.05	0.1053	<0.05	NS	<0.05
		RL	0.16	0.05	0.06	0.15			NS	
		RP	0.23	0.12	0.07	0.25			NS	
		RE	36.33^a^	8.53^b^	12.02	9.07			NS	
*Firmicutes*	*Acetitomaculum*	RS	2.05	1.14	1.35	2.87	0.0163	NS	NS	NS
		RL	0.42	0.46	0.18	0.37			NS	
		RP	1.81	1.27	0.61	1.11			<0.05	
		RE	1.24	1.25	0.85	1.16			NS	
	*Blautia*	RS	1.43	1.09	0.85	0.99	0.0113	NS	NS	NS
		RL	0.47	0.32	0.14	0.26			NS	
		RP	1.30	0.73	0.22	0.23			<0.05	
		RE	0.30	0.34	0.35	0.23			NS	
	*Butyrivibrio*	RS	0.16	0.12	0.20	0.30	0.0292	<0.05	<0.05	NS
		RL	0.14	0.09	0.10	0.31			NS	
		RP	0.03	0.07	0.03	0.10			NS	
		RE	2.19^b^	8.05^a^	6.33^b^	12.85^a^			NS	
	*Christensenellaceae R-7 group*	RS	1.52^b^	3.12^a^	4.39	3.73	0.0075	<0.01	<0.05	NS
		RL	0.40	1.10	0.73^b^	2.10^a^			NS	
		RP	0.73	1.76	0.61	1.41			NS	
		RE	0.43^b^	1.29^a^	1.19	1.30			NS	
	*Lachnospiraceae NK3A20 group*	RS	6.75^b^	16.31^a^	8.33	14.21	0.0911	<0.01	NS	<0.05
		RL	3.61	7.23	1.29	2.49			<0.05	
		RP	8.19^b^	19.67^a^	3.20	7.95			NS	
		RE	3.11^b^	10.55^a^	5.91	3.18			NS	
	*Lactobacillus*	RS	0.00	0.00	0.01	0.00	0.0002	NS	NS	NS
		RL	0.00	0.00	0.01	0.00			NS	
		RP	0.00	0.00	0.02	0.01			<0.05	
		RE	0.00	0.00	0.00	0.01			NS	
	*Quinella*	RS	4.41	1.90	10.59	7.00	0.2177	<0.01	<0.05	NS
		RL	11.24	1.14	36.00^a^	13.74^b^			<0.01	
		RP	16.07	9.46	49.58	29.88			<0.01	
		RE	1.67	0.86	6.01	7.73			<0.05	
	*Ruminiclostridium*	RS	0.56	0.72	1.05^a^	0.24^b^	0.0047	NS	NS	<0.01
		RL	0.23^b^	1.03^a^	0.43	0.16			NS	
		RP	0.32	0.30	0.17	0.06			<0.05	
		RE	0.23	0.28	0.35^a^	0.05^b^			NS	
	*Ruminococcaceae NK4A214 group*	RS	2.54^b^	3.79^a^	4.10	4.81	0.0449	<0.01	NS	NS
		RL	1.62	1.36	2.42^b^	4.27^a^			<0.05	
		RP	2.09^b^	3.87^a^	2.67	3.81			NS	
		RE	0.95	1.78	1.95	1.36			NS	
	*Ruminococcus*	RS	6.95	5.24	9.71	8.31	0.0695	NS	NS	NS
		RL	4.83	2.59	7.86	5.99			NS	
		RP	4.75	2.95	2.54	2.83			NS	
		RE	1.51	3.85	5.49	4.42			NS	
	*Selenomonas*	RS	1.51	0.47	0.55	0.83	0.0202	NS	NS	<0.05
		RL	2.54^a^	0.40^b^	0.56	1.21			NS	
		RP	0.94	0.23	0.09	0.27			NS	
		RE	0.35	0.18	0.24	0.09			NS	
	*Streptococcus*	RS	0.02	0.02	0.01	0.01	0.0005	NS	<0.01	NS
		RL	0.01	0.02	0.00	0.00			<0.05	
		RP	0.01	0.00	0.00	0.00			NS	
		RE	0.01	0.02	0.01	0.00			NS	
	*Succiniclasticum*	RS	0.93	1.54	0.47	0.76	0.0071	NS	<0.05	NS
		RL	0.72	0.41	0.19	0.35			NS	
		RP	1.30	1.70	0.15	0.39			<0.01	
		RE	0.59	0.91	0.33	1.00			NS	
*Bacteroidetes*	*Bacteroides*	RS	0.93	0.65	0.85	0.70	0.0073	NS	NS	NS
		RL	0.88	0.59	0.30	0.83			NS	
		RP	1.84	1.28	0.44	0.45			<0.05	
		RE	0.37	0.27	0.56	0.26			NS	
	*Porphyromonas*	RS	0.01	0.00	0.01	0.00	0.0010	NS	NS	NS
		RL	0.00	0.02	0.00	0.05			NS	
		RP	0.01	0.00	0.01	0.00			NS	
		RE	0.00	0.00	0.01	0.00			NS	
	*Prevotella*	RS	20.36	19.46	10.8	11.61	0.0590	NS	<0.01	NS
		RL	22.15	29.69	6.92	11.03			<0.01	
		RP	15.95	16.79	3.34	5.30			<0.01	
		RE	6.80	9.30	6.15	4.16			NS	
	*Prevotellaceae UCG-001*	RS	0.94	0.83	0.81	0.57	0.0365	NS	NS	NS
		RL	0.72	0.65	0.85	0.54			NS	
		RP	0.70	0.47	0.28	0.32			NS	
		RE	1.60^b^	4.26^a^	5.45	3.71			NS	
Fibrobacteres	*Fibrobacter*	RS	1.03	0.06	0.11	0.06	0.0239	NS	NS	NS
		RL	1.06	0.45	0.07	0.91			NS	
		RP	0.51	0.05	0.12	0.03			NS	
		RE	0.17	0.09	0.13	0.18			NS	
*Spirochaetae*	*Treponema*	RS	4.50	4.17	2.96	3.01	0.0104	NS	<0.05	NS
		RL	2.94	2.48	1.03	1.11			<0.05	
		RP	1.12	0.93	0.15	0.22			<0.01	
		RE	1.23	1.34	2.87^a^	0.90^b^			NS	
*Actinobacteria*	*Atopobium*	RS	1.94	2.11	3.73	1.86	0.0320	NS	NS	NS
		RL	0.72	0.62	0.30	0.22			NS	
		RP	3.12	1.92	0.85	0.80			<0.05	
		RE	0.83	0.85	1.55	0.74			NS	
Synergistetes	*Fretibacterium*	RS	6.42	4.14	13.28	8.66	0.1893	NS	NS	NS
		RL	8.90	3.88	20.99	18.57			<0.01	
		RP	10.53	6.06	17.35	22.41			NS	
		RE	1.61	1.80	6.41	7.51			<0.05	

a, b *Means within a row for diets that do not have a common superscript differ (P <0.05)*.

1 SEM for diet × age;

2 NS, not significant (P > 0.05);

3 C, Control diet;

4 *R, Rhubarb treatment*.

## Discussion

In the past few decades, research has been performed to explore the initial establishment of the bacterial microbiota in the rumen. By using a culture-dependent technique, Fonty et al. (1987) reported that the rumen of lamb was quickly colonized by an ample microbiota and dominated by the strictly anaerobes on day 2 after birth. The bacterial community was verified to colonize the rumen of goat on 0 d after birth (Jiao et al., 2015b), and more specifically, the existence of *Proteobacteria* (*Geobacter* spp.), and fibrolytic bacteria (*Fibrobacter succinogenes, Ruminococcus flavefaciens*, and *Prevotella ruminicola*) were detected in the rumen fluid and tissue 0–20 min after birth through real-time qPCR (Guzman et al., 2015). With the aid of amplicon sequencing, it was found that *Proteobacteria, Firmicutes*, and *Bacteroidetes* were the three predominant phyla in the rumen fluid in 1-day-old calves (Jami et al., 2013), 2-day-old dairy cattle (Rey et al., 2014), and 7-day-old goats (Wang et al., 2016). One significant similarity amongst those previous studies which employed culture-independent methods is that they were all conducted based on DNA extracted from the rumen contents. In contrast, the current research is the first to investigate the initial colonization and the subsequent evolution of the metabolically active bacterial microbiota, using RNA isolated across four fractions within the rumen of black goats. The taxonomic classification of bacteria in this study reveals that on the first day after birth, the potentially active bacterial populations in RL and RE were both dominated by the *Proteobacteria, Firmicutes*, and *Bacteroidetes*, which is in agreement with the previous findings. One of the major findings of the Global Rumen Census (Henderson et al., 2015) was that diet is the main driver of rumen microbial diversity and other studies have noted that individual animals have unique bacterial patterns (Jami and Mizrahi, 2012; Rey et al., 2014; Jiao et al., 2015a).

In the present study, individual variation existed in initial establishment as well as the subsequent evolution of the bacterial microbiome across four fractions. The initial colonization of microbiota in the rumen could stem from the microorganisms in the maternal vagina and skin, colostrum, or the surrounding environment (Dominguez-Bello et al., 2010; Hunt et al., 2011; Di Mauro et al., 2013). These sources and their actual effects which might vary amongst the individuals, and the host genetics could result in the individual variations in bacterial establishment and development (Mayer et al., 2012). Welkie et al. (2010) observed that Holstein cows fed the same ration possessed substantially different ruminal bacterial community compositions, but displayed similar ruminal pH, VFA profile, and performance (milk production and composition). Using a network bipartition approach for linking the microbial network to a bovine metabolic network, Taxis et al. (2015) reported that two bovine ruminal microbiotas which are rather dissimilar at the taxonomic level were considerably more similar at the metabolic network level. Therefore, it can be assumed that despite the individual variation in bacterial community composition under the same management, it is still possible for the goats to have similar metabolic and production performance in this study.

In the current study, the age of the goats increased along with the alterations of diets. From after birth to 15 d, the dam's milk was the unique source of feed for the goats. From 16 to 39 d, apart from the milk, the goats also had free access to solid starter. After goats were weaned off on 40 d, they were fed only solid starter. Therefore, as was illuminated in previous studies (Rey et al., 2014; Wang et al., 2016), the effect of age in this research actually involved the influence of diet. The Chao 1 index underwent an age-dependent rise both in RL and RE, which is consistent with the report of Wang et al. (2016) that the Chao1 richness of the methanogenic population in rumen fluid increased from 7 days to 2 years. Similarly, an age-dependent increment in Chao 1 of the bacteria community in RE was observed in our previous study (Wang et al., submitted). However, this pattern was not noted in RS and RP and it could be explained by the fact that the Chao 1 indices in these two fractions on 20 d were already relatively high, suggesting that the microbiota from 20 d on were more diverse than it was before. When compared with the microorganisms in RS and RL, knowledge on the microbial community associated with the RE are few. In this study, the Chao 1 richness of the bacterial population in RE was significantly lower than that of the other fractions on 50 d. An explanation for this phenomenon could be that RE locates at the border of host tissues and thus its interaction with different feed, diverse microbes, and complex microscale activities is limited compared to the microbiota in the other fractions, and the environmental heterogeneity could influence the microbial density, diversity, and composition (Horner-Devine et al., 2004; Liu et al., 2016). Moreover, as is reflected by the PCoA, samples in all the four fractions clustered according to different age groups, which is in line with the previous reports on the structure of bacterial population across different ages in rumen fluid (Jami et al., 2013; Rey et al., 2014) and rumen epithelium (Jiao et al., 2015a). This indicates that the structure of bacterial community progressively changed along with the shifts in diets over time, and the microbiota in each age group is distinct. As a strategy to accustom the suckling ruminants to a diet composed of forage and concentrates and lower the cost of production, early weaning and the related strategies have been studied intensively and regarded as an effective approach in regulating the microbial community and improving rumen fermentation (Windeyer et al., 2014; Guzman et al., 2016). In addition to the shift in diet structure and components, weaning could also cause psychological as well as physiological stress since the kids are no longer bred with their mothers (Lay et al., 1998; Loberg et al., 2008). In the present study, the bacterial communities in RS and RE on 38 d were separate from those on 41 d, implying that weaning altered the structure of the bacterial microflora in these two fractions. As is manifested in the PCoA, the structure of bacterial community in RE was always different from those in the other three fractions during rumen development, being supported by those researches in which discrepancies between the epithelial tissue-associated and the rumen contents-associated bacterial populations were observed (Sadet-Bourgeteau et al., 2010; Petri et al., 2013; Liu et al., 2016).

Previous studies performed have reported that *Proteobacteria, Firmicutes*, and *Bacteroidetes* were the three predominant phyla throughout different age groups (Jami et al., 2013; Rey et al., 2014; Jiao et al., 2015a; Wang et al., 2016). In contrast to the DNA-based 16S rRNA gene sequencing used in those above investigations, the RNA-derived amplicon sequencing could reflect the metabolically active microbes and their potential activities in rumen fermentation (Hugoni et al., 2013; Li et al., 2016). Similarly, results of the current study revealed that the potentially active bacterial microbiota in four fractions was dominated by the phyla *Proteobacteria, Firmicutes*, and *Bacteroidetes* regardless of different days. More specifically, the relative proportions of *Proteobacteria* in RE was consistently the highest compared to other three fractions, which is in agreement with the findings of Chen et al. (2011) and Liu et al. (2016). Rey et al. (2012) reported that the ruminal redox potential value in dairy calves at 1 d after birth was positive but then decreased dramatically and remained negative as the calves aged, and it was also noticed that the ruminal reducing power in lambs rose from 2 to 10 d after birth (Chaucheyras-Durand and Fonty, 2002), which together indicating the foundation and maintenance of a reducing environment in the rumen contents during rumen development. In the present study, the explanation for a greater abundance of *Proteobacteria* in RE might be that unlike the reducing conditions in the rumen contents, there is trace amount of oxygen in RE, and the phylum *Proteobacteria* includes many microaerophilic or facultative anaerobic bacteria which are capable of thriving in the environment with trace amount of oxygen (Liu et al., 2016). The consumption of oxygen by the epithelial microflora could contribute to the colonization and activities of the obligate anaerobes in the rumen (Jost et al., 2012). Moreover, RS, RL, and RP accommodated more *Bacteroidetes* than RE, being supported by previous report (Liu et al., 2016). It is believed that some species within the phylum *Bacteroidetes* could degrade soluble polysaccharides in plant cell wall, a great proportion of *Bacteroidetes* in the rumen contents could hence benefit the rumen fermentation of feed matter (Salyers et al., 1995; Power et al., 2014).

In this study, *Proteobacteria* was the most predominant phylum which occupied 70.25 and 89.05% of the bacterial community in RL and RE on 1 d, respectively. Nevertheless, substantial declines of its proportions in all the four fractions were subsequently observed, while the relative abundances of the phyla *Firmicutes* and *Bacteroidetes* increased in inverse proportion to that of the *Proteobacteria*, being the most and second dominant, respectively. This is partly in line with the findings of a few parallel reports (Jami et al., 2013; Rey et al., 2014; Jiao et al., 2015a). Furthermore, since most of the genera detected within the phyla *Firmicutes* and *Bacteroidetes* in current research are anaerobes, the above phenomenon could be defined as a transition from an aerobic or facultative anaerobic to an exclusively anaerobic environmental niche, reflecting the rapid change of the bacterial microflora during normal development of rumen. Meale et al. (2016) observed the increases in the abundances of *Proteobacteria* and *Firmicutes*, but the decrease in that of *Bacteroidetes* in the rumen fluid of dairy calves post weaning vs. before weaning. By contrast, result of the present study shows that on 41 d the ratios of *Proteobacteria* in four fractions increased, however most of the proportions of *Firmicutes* and *Bacteroidetes* were reduced compared to those on 38 d. This inconsistency could result from the differences in animal species or the methods of weaning used.

In contrast to the phyla, the composition and its dynamics of the bacterial microbiota at genus level in the present sequencing-based study were much more diverse and complex. On 1 d after birth, the most prevalent phylum *Proteobacteria* was mainly dominated by the facultative anaerobic genus *Mannheimia* which underwent dramatical decline afterwards. Conversely, different types of increases in the relative proportions of the genera *Ruminobacter* and *Suttonella* within the phylum *Proteobacteria* and most of the genera in *Firmicutes* and Bacteroides were observed. As the age increased and the goats were successively supplemented with solid feed and weaned, the bacterial populations across the four fractions became mainly predominated by the genera *Quinella, Prevotella, Fretibacterium, Ruminococcus, Lachnospiraceae NK3A20 group*, and *Atopobium*. Some members of the genus *Quinella* were found to possess a metabolism of utilizing lactate similar to *Selenomonas ruminantium* (Krumholz et al., 1993; Belenguer et al., 2010), while the colonization of the genus *Fretibacterium* were previously reported in human oral cavity (Szafranski et al., 2015). The functional roles of these two genera in rumen fermentation during rumen development are yet to be explored and examined. It has been observed that some ruminal species of genus *Prevotella* have the capability to utilize starches, simple sugars, and other non-cellulosic polysaccharides as energy sources (Purushe et al., 2010). Within the genus *Ruminococcus, R. flavefaciens* and *R. albus* are two major cellulolytic species frequently found in the adult rumen (Flint and Bayer, 2008).

In the current study, the relative abundances of *Ruminococcus* in four fractions remained low on 1 and 10 d but started to increase from 20 d on, which is in accordance of the initial intake of solid feed on 15 d. Since it was reported that strains of the family *Lachnospiraceae* detected in the rumen and are potentially able to biohydrogenate fatty acids (Boeckaert et al., 2009), the *Lachnospiraceae NK3A20 group* found in this research might possess similar potential. Members of the genus *Atopobium* are Gram-positive and anaerobic bacteria which together consistute a distinct group (Coriobacteria) within the Actinomycetes (Mao et al., 2013), Harmsen et al. (2000) found that the growth of the *Atopobium* was elevated by sugars. Similarly in present study, the abundances of *Atopobium* in four fractions were enhanced as the starter concentrate was introduced. Furthermore, the proportions of the genera *Selenomonas, Streptococcus*, and *Fibrobacter* across fractions stayed at relatively low level during rumen development in this study, being supported by the finding that these three major genera were identified with relative abundances <1% in the rumen of adult cows (Zened et al., 2013). Henderson et al. (2015) suggested that the pivotal function of *Fibrobacter* could still be achieved despite of its low relative abundance. The prevalence of the genera *Butyrivibrio* and *Campylobacter* in RE was reported in previous researches (Jiao et al., 2015a; Liu et al., 2016), higher proportions of these two genera were also noted in the current study. It is hypothesized that, as butyrate producers, these genera could release butyrate close to the epithelium and thus improve butyrate bioavailability for the host, which might be beneficial to the proliferation of rumen and reticulum epithelium (Siavoshian et al., 2000). Jiao et al. (2015a) proposed that *Butyrivibrio* might participate in the metabolism of ammonia and volatile fatty acid, and the anatomic development of the rumen. Moreover, it was reported that specific symbiotic microbes could influence the host immune function by regulating host immune responses through proinflammatory and anti-inflammatory pathways, and the epithelial-cell-mediated signals as well (Klaenhammer et al., 2012; Malmuthuge et al., 2014).

In the present study we found that after the introduction of solid starter, the relative abundance of *Campylobacter* in RE was constantly higher than the other three fractions. The human foodborne pathogen *Campylobacter* has been previously detected in rumen contents and at levels of up to 30.00% in RE samples (Jiao et al., 2015a). The presence of increased levels of *Campylobacter* in RE samples is in agreement with its microaerophilic metabolism, and may be an opportunistic, transient member of the RE microbial community. In the present study, when compared to the other three fractions, the abundances of *Suttonella* and *Prevotellaceae UCG-001* in RE were higher, while less relative ratios of *Quinella* and *Prevotella* were presented in RE. It is also remarked that RS harbored the most *Blautia* than other three fractions. These discrepancies amongst fractions necessitate further investigations to explore the mechanisms in the distributions of bacterial populations across different fractions, and deepen the understandings on the commensal interactions between the epimural microflora and the host.

Effects of rhubarb treatment on rumen fermentation have been examined in a few investigations *in vitro* and *in vivo*, indicating that rhubarb might manipulate rumen fermentation by reducing methane production and the acetate: propionate ratio without negative side effects, such as suppressing feed intake or digestibility (Bodas et al., 2008; García-González et al., 2010, 2012). Recently, it was found that the relative abundances of *Prevotella* and *Lactobacillus* were improved after the treatment of rhubarb (Kim et al., 2016). By contrast, result of the present study shows no difference in the abundances of *Lactobacillus* between the control diet and the rhubarb treatment, and there were only numerical increases in the proportions of *Prevotella* in rhubarb treatment compared to the control. Further, the addition of rhubarb enhanced the relative abundances of the genera *Christensenellaceae R-7 group, Lachnospiraceae NK3A20 group*, and *Ruminococcaceae NK4A214 group* to different extents, but decreased the proportions of *Quinella* spp. in four fractions. Further investigations are warranted to explore the mechanisms of rhubarb in manipulating the rumen bacterial population and its connection with the methanogen community and methanogenesis. Moreover, in comparison with the 50 d, the effects of rhubarb on the bacterial community structure as well as the relative abundances of bacterial genera were less significant on 60 d in the present study. This phenomenon was also noted in our previous study on the methanogen community during rumen development (Wang et al., submitted), and it could be probably ascribed to the potential rumen microbial adaptation (Hart et al., 2008; García-González et al., 2010, 2012).

This study observed individual variations in the metabolically active bacterial community composition, and the rapidly changing bacterial microbiota across fractions in response to the change of diet and age during rumen development. The diversities and structures of bacterial population, and the distributions of diverse bacteria differed across the four fractions during the normal development of rumen, implying that the disparity amongst these four fractions should be taken into account when exploring the overall bacterial community and formulating strategies for microbial programming. The interactions within the complex bacterial microbiota during rumen development and the relevant factors and mechanisms require further investigations. This study provides information on the development of the rumen bacterial community and the related modulation. Future research should try to explain the interactions of the anatomical, functional, and microbial development, as well as the impact of manipulation during early life on ruminant production in the long term.

## Author contributions

ZW, ZT, and RF designed the research; ZW, JJ, MW, ST, and CZ conducted the research; ZW and CE analyzed the data; ZW, RF, and ZT wrote the paper. All authors approved the final manuscript.

## Funding

This study was supported by the grants from the National Natural Science Foundation of China (Grant No. 31320103917, 31561143009), “Strategic Priority Research Program-Climate Change: Carbon Budget and Relevant Issues” (Grant No.XDA05020700), and Hunan Provincial Creation Development Project (Grant No. 2013TF3006). This study was also supported by the Open Foundation of Key Laboratory of Agro-ecological Processes in Subtropical Region, Institute of Subtropical Agriculture, Chinese Academy of Sciences (Grant No. ISA2016301), and the MOE-AAFC PhD Research Program of the China Scholarship Council.

### Conflict of interest statement

The authors declare that the research was conducted in the absence of any commercial or financial relationships that could be construed as a potential conflict of interest.
