# Supplementary material for: Changes in Metabolically Active Bacterial Community during Rumen Development, and Their Alteration by Rhubarb Root Powder Revealed by 16S rRNA Amplicon Sequencing
Source: Front Microbiol. 2017 Feb 7;8:159. doi: 10.3389/fmicb.2017.00159 (PMC5293741; doi:10.3389/fmicb.2017.00159)
Supplement: Supplementary file 1 [file Table1.PDF]

Supplementary Material

Changes in metabolically active bacterial community during rumen development, and their alteration by rhubarb root powder revealed by 16S rRNA amplicon sequencing

Zuo Wang [1,2,3], Chijioke Elekwachi [3], Jinzhen Jiao [1], Min Wang [1], Shaoxun Tang [1], Chuanshe Zhou [1], Zhiliang Tan [1*], and Robert J. Forster [3*]

[1] Key Laboratory for Agro-Ecological Processes in Subtropical Region, Hunan Research Center of Livestock & Poultry Sciences, South-Central Experimental Station of Animal Nutrition and Feed Science in Ministry of Agriculture, Institute of Subtropical Agriculture, Chinese Academy of Sciences, Changsha, Hunan 410125, China

[2] University of Chinese Academy of Sciences, Beijing 100049, China

[3] Lethbridge Research and Development Centre, Agriculture and Agri-Food Canada, Lethbridge, AB T1J 4B1, Canada

*** Correspondence:**

Zhiliang Tan; Robert J. Forster

zltan@isa.ac.cn; robert.forster@agr.gc.ca

Supplementary Tables

Table S1. Dual index amplicon primers for bacteria

Name	Sequence	Barcode
Bact_seq_R1	TATGGTAATTGTACTCCTACGGGNGGCWGCAG	
Bact_seq_R2	AGTCAGTCAGCCGGACTACHVGGGTATCTAAT	
Bact_Index	ATTAGATACCCBDGTAGTCCGGCTGACTGACT	
FB501	AATGATACGGCGACCACCGAGATCTACACAACAACCGTATGGTAATTGTACTCCTACGGGNGGCWGCAG	AACAACCG
FB502	AATGATACGGCGACCACCGAGATCTACACACAAGAGGTATGGTAATTGTACTCCTACGGGNGGCWGCAG	ACAAGAGG
FB503	AATGATACGGCGACCACCGAGATCTACACCTTCTCGATATGGTAATTGTACTCCTACGGGNGGCWGCAG	CTTCTCGA
FB504	AATGATACGGCGACCACCGAGATCTACACCGACAAGATATGGTAATTGTACTCCTACGGGNGGCWGCAG	CGACAAGA
FB505	AATGATACGGCGACCACCGAGATCTACACGTGGTCTTTATGGTAATTGTACTCCTACGGGNGGCWGCAG	GTGGTCTT
FB506	AATGATACGGCGACCACCGAGATCTACACTCCTTCACTATGGTAATTGTACTCCTACGGGNGGCWGCAG	TCCTTCAC
FB507	AATGATACGGCGACCACCGAGATCTACACAAGACTGGTATGGTAATTGTACTCCTACGGGNGGCWGCAG	AAGACTGG
FB508	AATGATACGGCGACCACCGAGATCTACACTTAACGCGTATGGTAATTGTACTCCTACGGGNGGCWGCAG	TTAACGCG

FB701	CAAGCAGAAGACGGCATACGAGATAACACGACAGTCAGTCAGCCGGACTACHVGGGTATCTAAT	AACACGAC
FB702	CAAGCAGAAGACGGCATACGAGATAAGAGGCAAGTCAGTCAGCCGGACTACHVGGGTATCTAAT	AAGAGGCA
FB703	CAAGCAGAAGACGGCATACGAGATTTCTGAGGAGTCAGTCAGCCGGACTACHVGGGTATCTAAT	TTCTGAGG
FB704	CAAGCAGAAGACGGCATACGAGATCGACTCTTAGTCAGTCAGCCGGACTACHVGGGTATCTAAT	CGACTCTT
FB705	CAAGCAGAAGACGGCATACGAGATGAACTGCTAGTCAGTCAGCCGGACTACHVGGGTATCTAAT	GAACTGCT
FB706	CAAGCAGAAGACGGCATACGAGATGTGTAACCAGTCAGTCAGCCGGACTACHVGGGTATCTAAT	GTGTAACC
FB707	CAAGCAGAAGACGGCATACGAGATTCGAACCAAGTCAGTCAGCCGGACTACHVGGGTATCTAAT	TCGAACCA
FB708	CAAGCAGAAGACGGCATACGAGATCAACCTAGAGTCAGTCAGCCGGACTACHVGGGTATCTAAT	CAACCTAG
FB709	CAAGCAGAAGACGGCATACGAGATTGTCAGTGAGTCAGTCAGCCGGACTACHVGGGTATCTAAT	TGTCAGTG
FB710	CAAGCAGAAGACGGCATACGAGATGATCGGTAAGTCAGTCAGCCGGACTACHVGGGTATCTAAT	GATCGGTA
FB711	CAAGCAGAAGACGGCATACGAGATAGCATGGAAGTCAGTCAGCCGGACTACHVGGGTATCTAAT	AGCATGGA
FB712	CAAGCAGAAGACGGCATACGAGATTGAGGATGAGTCAGTCAGCCGGACTACHVGGGTATCTAAT	TGAGGATG
FC501	AATGATACGGCGACCACCGAGATCTACACAGCTTACCTATGGTAATTGTACTCCTACGGGNGGCWGCAG	AGCTTACC
FC502	AATGATACGGCGACCACCGAGATCTACACATACGTGCTATGGTAATTGTACTCCTACGGGNGGCWGCAG	ATACGTGC
FC503	AATGATACGGCGACCACCGAGATCTACACCAGTAAGGTATGGTAATTGTACTCCTACGGGNGGCWGCAG	CAGTAAGG
FC504	AATGATACGGCGACCACCGAGATCTACACCCATACTGTATGGTAATTGTACTCCTACGGGNGGCWGCAG	CCATACTG
FC505	AATGATACGGCGACCACCGAGATCTACACGGCTCAATTATGGTAATTGTACTCCTACGGGNGGCWGCAG	GGCTCAAT
FC506	AATGATACGGCGACCACCGAGATCTACACGATGACTCTATGGTAATTGTACTCCTACGGGNGGCWGCAG	GATGACTC
FC507	AATGATACGGCGACCACCGAGATCTACACGCAAGCAATATGGTAATTGTACTCCTACGGGNGGCWGCAG	GCAAGCAA
FC508	AATGATACGGCGACCACCGAGATCTACACTGTGTGCTTATGGTAATTGTACTCCTACGGGNGGCWGCAG	TGTGTGCT
FC701	CAAGCAGAAGACGGCATACGAGATACAATGCCAGTCAGTCAGCCGGACTACHVGGGTATCTAAT	ACAATGCC
FC702	CAAGCAGAAGACGGCATACGAGATGGACGTATAGTCAGTCAGCCGGACTACHVGGGTATCTAAT	GGACGTAT
FC703	CAAGCAGAAGACGGCATACGAGATCTTCCTTCAGTCAGTCAGCCGGACTACHVGGGTATCTAAT	CTTCCTTC
FC704	CAAGCAGAAGACGGCATACGAGATGAGAGAGTAGTCAGTCAGCCGGACTACHVGGGTATCTAAT	GAGAGAGT
FC705	CAAGCAGAAGACGGCATACGAGATTTGCGGAAAGTCAGTCAGCCGGACTACHVGGGTATCTAAT	TTGCGGAA
FC706	CAAGCAGAAGACGGCATACGAGATAATCGCCTAGTCAGTCAGCCGGACTACHVGGGTATCTAAT	AATCGCCT
FC707	CAAGCAGAAGACGGCATACGAGATCGGAATTGAGTCAGTCAGCCGGACTACHVGGGTATCTAAT	CGGAATTG
FC708	CAAGCAGAAGACGGCATACGAGATCGTGCATAAGTCAGTCAGCCGGACTACHVGGGTATCTAAT	CGTGCATA
FC709	CAAGCAGAAGACGGCATACGAGATACACTCAGAGTCAGTCAGCCGGACTACHVGGGTATCTAAT	ACACTCAG
FC710	CAAGCAGAAGACGGCATACGAGATCACTAGTCAGTCAGTCAGCCGGACTACHVGGGTATCTAAT	CACTAGTC
FC711	CAAGCAGAAGACGGCATACGAGATTCCGTAGTAGTCAGTCAGCCGGACTACHVGGGTATCTAAT	TCCGTAGT
FC712	CAAGCAGAAGACGGCATACGAGATGACTTGAGAGTCAGTCAGCCGGACTACHVGGGTATCTAAT	GACTTGAG
