# Supplementary material for: Changes in Metabolically Active Bacterial Community during Rumen Development, and Their Alteration by Rhubarb Root Powder Revealed by 16S rRNA Amplicon Sequencing
Source: Front Microbiol. 2017 Feb 7;8:159. doi: 10.3389/fmicb.2017.00159 (PMC5293741; doi:10.3389/fmicb.2017.00159)
Supplement: Supplementary file 2 [file Table2.PDF]

Supplementary Material

Changes in metabolically active bacterial community during rumen development, and their alteration by rhubarb root powder revealed by 16S rRNA amplicon sequencing

Zuo Wang [1,2,3], Chijioke Elekwachi [3], Jinzhen Jiao [1], Min Wang [1], Shaoxun Tang [1], Chuanshe Zhou [1], Zhiliang Tan [1*], and Robert J. Forster [3*]

[1] Key Laboratory for Agro-Ecological Processes in Subtropical Region, Hunan Research Center of Livestock & Poultry Sciences, South-Central Experimental Station of Animal Nutrition and Feed Science in Ministry of Agriculture, Institute of Subtropical Agriculture, Chinese Academy of Sciences, Changsha, Hunan 410125, China

[2] University of Chinese Academy of Sciences, Beijing 100049, China

[3] Lethbridge Research and Development Centre, Agriculture and Agri-Food Canada, Lethbridge, AB T1J 4B1, Canada

*** Correspondence:**

Zhiliang Tan; Robert J. Forster

zltan@isa.ac.cn; robert.forster@agr.gc.ca

Supplementary Tables

Table S2. Summary of sequencing data

Note: All sequenced samples had a RIN value ⩾8.							
Sample #	Bacterial sequences	observed_otus	goods_coverage	shannon	chao1	simpson	PD_whole_tree
RS20.1	15886	3530	0.825380838	8.244469	15549.2	0.97221695	130.22543
RS20.5	15876	3533	0.827853364	8.577085	14260.8	0.98602056	128.76809
RS20.6	15863	3475	0.821660468	8.414795	17510.8	0.98650558	120.40105
RS38.1	15883	3898	0.807278222	8.452493	16729	0.96856536	139.63186
RS38.2	15882	4528	0.773013474	9.181586	20850.1	0.98551231	166.96737
RS38.3	15860	4168	0.797351828	9.089199	17684.5	0.98333079	153.90505
RS38.4	15860	4477	0.770239596	9.133444	23550.4	0.98492495	157.61465
RS41.1	11016	3384	0.760348584	8.928128	13569.6	0.98268136	121.07475
RS41.3	15872	4778	0.755103327	9.635501	24243.1	0.99336136	180.83624
RS41.4	15868	4535	0.769284094	9.000236	22027.5	0.97161889	172.38644

RS50.1	15830	4579	0.762665824	9.005487	23596.9	0.98351015	170.6766
RS50.2	15818	4433	0.768870907	8.965952	23577.2	0.98630791	159.08629
RS50.3	15857	4387	0.773223182	8.845145	21308	0.98458902	152.31464
RS50.4	15798	4606	0.761298899	9.343071	24406.4	0.98959568	172.07984
RS60.1	15847	4286	0.780021455	8.801523	21791.3	0.98422185	156.52097
RS60.2	10358	3291	0.73344275	8.729835	20150.2	0.9827002	129.97453
RS60.3	15294	4620	0.752582712	9.162052	24069.6	0.98591083	177.33101
RS60.4	15888	4968	0.741565962	9.439766	27204.3	0.99040353	188.64454
RS50R.1	9909	3066	0.733070946	8.532489	20997.7	0.97731631	111.23201
RS50R.2	9911	3301	0.719503582	9.248373	22909.2	0.991211	131.83486
RS50R.3	9887	3241	0.727318701	9.33937	21681.9	0.99150329	125.23137
RS60R.1	9921	3312	0.730067534	9.322162	15672.4	0.99111831	127.21003
RS60R.2	9915	3379	0.721835603	9.210937	18647.7	0.98305993	128.52168
RS60R.3	9907	3423	0.714040577	8.988007	20137.7	0.97932887	141.31249
L1.4	14871	2647	0.866451483	5.791957	8826.01	0.85170602	70.73994
L1.6	14872	2714	0.864779451	5.811754	8281.64	0.8622137	70.21432
L1.7	14857	3286	0.824594467	7.16082	12289.5	0.94636817	87.35863
L1.8	14824	3302	0.813005936	6.979915	15731.1	0.95138214	90.79405
L10.1	14793	3047	0.831271547	7.28828	15115.8	0.9614189	109.89586
L10.3	14736	2927	0.831501086	6.664529	16933.4	0.93643	101.94116
L10.4	14793	3373	0.811600081	7.603901	16623.1	0.96102982	116.83972
L10.5	14862	3153	0.828556049	6.215773	13261.7	0.82183469	104.44112
L10.6	14763	2645	0.851588431	6.111309	12897.8	0.91769936	96.30821
L10.7	14657	3016	0.825475882	6.795787	16815.2	0.93485562	106.20842
L20.1	14891	3462	0.815190383	8.230785	15109.3	0.9781179	121.19882
L20.2	14829	3327	0.82217277	7.584879	14431	0.95418397	124.12566
L20.3	14828	3413	0.81379822	7.81757	15987.9	0.96531127	124.09845
L20.4	14849	3680	0.791029699	7.775379	20746.5	0.9606539	138.64579
L20.5	14869	4069	0.776447643	8.729853	20408.7	0.98328565	140.55815
L20.6	14889	3492	0.800792531	7.8641	21080.4	0.97413566	131.17853
L38.1	14905	3489	0.813552499	8.162795	15665.8	0.96023432	125.49249
L38.2	14858	4008	0.789069861	8.562518	17064.9	0.9686928	143.46565
L38.3	14877	3911	0.792364052	8.548653	19446.6	0.96415315	134.96163
L38.4	14840	3669	0.796832884	8.046867	19176.2	0.95138848	130.92758
L41.1	9937	3152	0.75535876	9.367399	13302.1	0.98937417	124.333
L41.3	14878	4412	0.756822154	9.078756	23268.3	0.98183297	167.03788
L41.4	14861	4188	0.772424467	8.209734	19273.1	0.92526062	152.17234
L50.1	14854	3664	0.802410125	8.109504	16791	0.96987891	135.56937
L50.2	14870	4065	0.773907196	8.300428	20731.2	0.96792759	139.94384
L50.3	14870	4295	0.75870881	9.162373	25674	0.9910981	146.02248
L50.4	14837	4486	0.75001685	9.297889	24651.6	0.99061948	161.05198
L60.1	14873	3280	0.829019028	6.420954	13255.8	0.80521871	116.07239
L60.2	14844	3945	0.777957424	7.251399	21461.6	0.90701323	138.28287

L60.3	14869	4192	0.76669581	8.294851	23288	0.96678303	154.35996
L60.4	14875	4516	0.745882353	8.78107	23977.3	0.98302608	165.95194
L50R.1	9912	2578	0.78319209	7.754024	14414	0.96264574	92.49556
L50R.2	9920	2802	0.765927419	8.481	15219.9	0.98343498	110.46298
L50R.3	9901	3175	0.735077265	8.993093	17326.2	0.98791887	120.51879
L60R.1	9911	3199	0.732923015	9.118196	18973.7	0.98672024	123.42837
L60R.2	9898	2810	0.772277228	8.778696	14095	0.98757841	108.15187
L60R.3	2614	1632	0.437643458	9.326291	14186.8	0.98700014	75.87712
RP20.2	14875	3615	0.809680672	7.935805	14899.1	0.95853076	123.19172
RP20.3	14831	3408	0.816263232	7.329101	15227.9	0.94793697	120.08494
RP20.5	14882	3834	0.791425884	8.567693	17672.7	0.9782287	134.14179
RP20.6	14840	3623	0.79925876	8.1415	18166.4	0.97665843	123.6156
RP38.1	14888	3698	0.80413756	7.917768	15278.6	0.94985408	124.04433
RP38.2	14906	3908	0.794981886	7.481553	15877.3	0.88566065	125.19657
RP38.3	1778	954	0.538245219	8.716923	6659.25	0.99205892	50.75077
RP41.1	8083	2662	0.73574168	8.023415	11746.4	0.95435411	102.46463
RP41.3	14902	3913	0.785666354	8.563781	20101	0.98092444	140.63701
RP41.4	14836	4057	0.779724993	8.082557	18926.9	0.92054472	145.85935
RP50.1	14863	3788	0.79896387	8.022886	16115.6	0.94505252	134.04965
RP50.2	14889	3894	0.78232252	7.827072	19852.7	0.95109149	128.20965
RP50.3	14873	4067	0.770994419	8.557187	22244.8	0.97999713	135.51041
RP50.4	14840	4239	0.763207547	8.737021	22392.9	0.9819873	150.03133
RP60.1	14898	3005	0.847160693	5.297294	10243.1	0.66914462	108.59745
RP60.2	14877	3680	0.79491833	6.760012	17908.7	0.88194387	128.32378
RP60.3	14892	3341	0.820306205	6.864169	13806.4	0.92005764	119.25369
RP60.4	14866	4030	0.776200726	7.947893	19928.9	0.96385269	136.72054
RP50R.1	9939	2872	0.760237448	8.108955	15320	0.96174136	108.438
RP50R.2	9917	3125	0.7358072	8.657473	18935.6	0.98142572	122.65275
RP50R.3	9907	2940	0.753810437	8.889686	17303	0.98880189	108.83734
RP60R.1	9923	3194	0.728307971	8.436912	19412.1	0.96772107	118.78551
RP60R.2	9920	3096	0.751209677	8.959401	14895.5	0.98668018	117.09046
RP60R.3	9924	2547	0.785570335	6.530347	12386.7	0.89380635	97.09803
RE1.3	6461	1449	0.824949698	6.032275	5467.96	0.89715697	51.81897
RE1.4	7806	1695	0.831411735	5.444265	6065.05	0.80735527	52.91194
RE1.6	9913	2275	0.815595682	5.740066	9232.83	0.82971327	70.33048
RE1.8	6800	1837	0.766764706	6.248427	11287.4	0.89102855	61.93094
RE10.2	9890	2661	0.781193124	5.766087	10930.8	0.71442999	76.38457
RE10.3	9834	2342	0.801810047	5.651053	10779	0.81532924	70.72266
RE10.4	9875	2237	0.808708861	5.333715	10379.5	0.74591259	74.7146
RE10.6	8358	1771	0.821966978	6.135844	8817.68	0.92036265	61.44645
RE20.1	3051	1006	0.728285808	7.521331	5463.22	0.97209753	46.17794
RE20.2	9121	2164	0.800350839	6.511019	11742.7	0.92458639	75.92516
RE20.3	6752	1912	0.763477488	7.124123	9364.67	0.94457373	67.86504

RE38.1	8401	2314	0.781216522	8.143965	11016.1	0.97473134	81.64156
RE38.3	9874	2523	0.799270812	7.841396	10161.8	0.97026941	84.20391
RE38.4	9871	2523	0.789889576	7.461429	12521.6	0.95039294	86.37227
RE41.1	9882	3261	0.729204614	8.215288	14696	0.94733844	109.91054
RE41.2	6771	2178	0.726037513	7.806783	12413.6	0.95858398	79.96949
RE41.3	7964	2555	0.737317931	8.806416	14912	0.98599385	94.26802
RE50.1	9713	1953	0.84865644	5.52654	6730.5	0.72475776	59.83597
RE50.2	5560	1614	0.753956835	6.896265	9674.59	0.92484945	59.57046
RE50.3	9841	2633	0.771771161	7.560605	17290.8	0.95753481	91.03314
RE50.4	9827	2496	0.794850921	7.040891	11404.4	0.8802045	84.25287
RE60.1	9821	2667	0.772019143	7.867018	15648.6	0.96733262	90.78919
RE60.2	9863	2561	0.784345534	7.903163	14215.6	0.97353604	90.05316
RE60.3	5616	1871	0.728098291	8.521023	9906.18	0.98614168	72.33229
RE60.4	9861	2628	0.775986208	7.853175	15739.5	0.96924897	88.0547
RE50R.1	9339	2118	0.814327016	7.485507	11279.7	0.96742177	76.50015
RE50R.2	2497	843	0.725670805	7.096914	4339.57	0.94610385	41.55346
RE50R.3	9885	2523	0.798482549	8.357448	11415.5	0.9834496	96.2048
RE60R.1	3232	1009	0.742883663	7.150271	5319.81	0.95965948	46.6137
RE60R.2	9175	2420	0.786158038	7.593877	11804.1	0.96412183	84.8036
RE60R.3	9889	2668	0.785721509	8.180084	12868.1	0.96938593	104.02883
