# Supplementary material for: Changes in Metabolically Active Bacterial Community during Rumen Development, and Their Alteration by Rhubarb Root Powder Revealed by 16S rRNA Amplicon Sequencing
Source: Front Microbiol. 2017 Feb 7;8:159. doi: 10.3389/fmicb.2017.00159 (PMC5293741; doi:10.3389/fmicb.2017.00159)
Supplement: Supplementary file 3 [file Image1.PDF]

Supplementary Material

Changes in metabolically active bacterial community during rumen development, and their alteration by rhubarb root powder revealed by 16S rRNA amplicon sequencing

Zuo Wang [1,2,3], Chijioke Elekwachi [3], Jinzhen Jiao [1], Min Wang [1], Shaoxun Tang [1], Chuanshe Zhou [1], Zhiliang Tan [1*], and Robert J. Forster [3*]

[1] Key Laboratory for Agro-Ecological Processes in Subtropical Region, Hunan Research Center of Livestock & Poultry Sciences, South-Central Experimental Station of Animal Nutrition and Feed Science in Ministry of Agriculture, Institute of Subtropical Agriculture, Chinese Academy of Sciences, Changsha, Hunan 410125, China

[2] University of Chinese Academy of Sciences, Beijing 100049, China

[3] Lethbridge Research and Development Centre, Agriculture and Agri-Food Canada, Lethbridge, AB T1J 4B1, Canada

*** Correspondence:**

Zhiliang Tan; Robert J. Forster

zltan@isa.ac.cn; robert.forster@agr.gc.ca

Supplementary Figures

Figure S1. Management of goats and sampling from four fractions during rumen development.

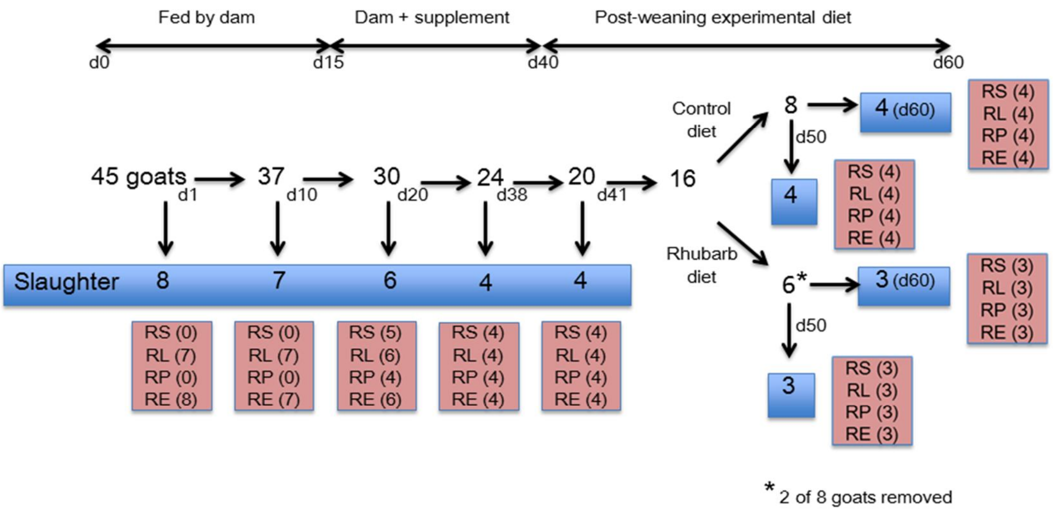

Fed by dam
Dam + supplement
Post-weaning experimental diet
d0
d15
d40
d60
45 goats
d1
37
d10
30
d20
24
d38
20
d41
16
Slaughter
8
7
6
4
4
RS (0) RL (7) RP (0) RE (8)
RS (0) RL (7) RP (0) RE (7)
RS (5) RL (6) RP (4) RE (6)
RS (4) RL (4) RP (4) RE (4)
RS (4) RL (4) RP (4) RE (4)
Control diet
8
4 (d60)
RS (4) RL (4) RP (4) RE (4)
d50
4
RS (4) RL (4) RP (4) RE (4)
Rhubarb diet
6*
3 (d60)
RS (3) RL (3) RP (3) RE (3)
d50
3
RS (3) RL (3) RP (3) RE (3)
* 2 of 8 goats removed
