# Supplementary material for: Changes in Metabolically Active Bacterial Community during Rumen Development, and Their Alteration by Rhubarb Root Powder Revealed by 16S rRNA Amplicon Sequencing
Source: Front Microbiol. 2017 Feb 7;8:159. doi: 10.3389/fmicb.2017.00159 (PMC5293741; doi:10.3389/fmicb.2017.00159)
Supplement: Supplementary file 5 [file Image3.PDF]

Supplementary Material

Changes in metabolically active bacterial community during rumen development, and their alteration by rhubarb root powder revealed by 16S rRNA amplicon sequencing

Zuo Wang [1,2,3], Chijioke Elekwachi [3], Jinzhen Jiao [1], Min Wang [1], Shaoxun Tang [1], Chuanshe Zhou [1], Zhiliang Tan [1*], and Robert J. Forster [3*]

[1] Key Laboratory for Agro-Ecological Processes in Subtropical Region, Hunan Research Center of Livestock & Poultry Sciences, South-Central Experimental Station of Animal Nutrition and Feed Science in Ministry of Agriculture, Institute of Subtropical Agriculture, Chinese Academy of Sciences, Changsha, Hunan 410125, China

[2] University of Chinese Academy of Sciences, Beijing 100049, China

[3] Lethbridge Research and Development Centre, Agriculture and Agri-Food Canada, Lethbridge, AB T1J 4B1, Canada

*** Correspondence:**

Zhiliang Tan; Robert J. Forster

zltan@isa.ac.cn; robert.forster@agr.gc.ca

Supplementary Figures

Figure S3. Composition of bacterial communities at phylum level in RS (A), RL (B), RP (C), and RE (D) during rumen development.

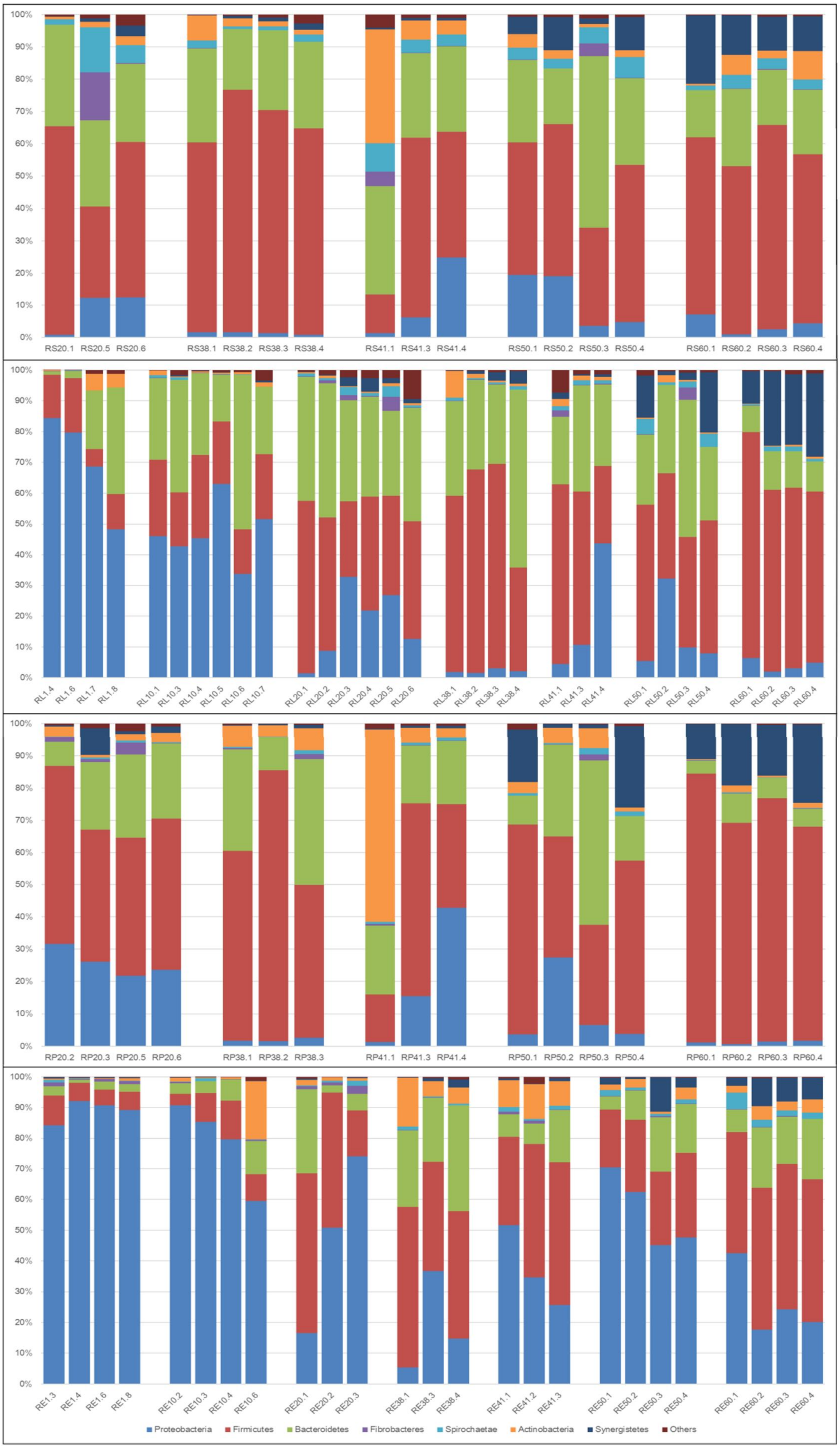
A
100%
90%
80%
70%
60%
50%
40%
30%
20%
10%
0%
RS20.1 RS20.5 RS20.6 RS38.1 RS38.2 RS38.3 RS38.4 RS41.1 RS41.3 RS41.4 RS50.1 RS50.2 RS50.3 RS50.4 RS60.1 RS60.2 RS60.3 RS60.4
B
RL1.4 RL1.6 RL1.7 RL1.8 RL10.1 RL10.3 RL10.4 RL10.5 RL10.6 RL10.7 RL20.1 RL20.2 RL20.3 RL20.4 RL20.5 RL20.6 RL38.1 RL38.2 RL38.3 RL38.4 RL41.1 RL41.3 RL41.4 RL50.1 RL50.2 RL50.3 RL50.4 RL60.1 RL60.2 RL60.3 RL60.4
C
RP20.2 RP20.3 RP20.5 RP20.6 RP38.1 RP38.2 RP38.3 RP41.1 RP41.3 RP41.4 RP50.1 RP50.2 RP50.3 RP50.4 RP60.1 RP60.2 RP60.3 RP60.4
D
RE1.3 RE1.4 RE1.6 RE1.8 RE10.2 RE10.3 RE10.4 RE10.6 RE20.1 RE20.2 RE20.3 RE38.1 RE38.3 RE38.4 RE41.1 RE41.2 RE41.3 RE50.1 RE50.2 RE50.3 RE50.4 RE60.1 RE60.2 RE60.3 RE60.4
Proteobacteria
Firmicutes
Bacteroidetes
Fibrobacteres
Spirochaetae
Actinobacteria
Synergistetes
Others
