# Supplementary material for: Changes in Metabolically Active Bacterial Community during Rumen Development, and Their Alteration by Rhubarb Root Powder Revealed by 16S rRNA Amplicon Sequencing
Source: Front Microbiol. 2017 Feb 7;8:159. doi: 10.3389/fmicb.2017.00159 (PMC5293741; doi:10.3389/fmicb.2017.00159)
Supplement: Supplementary file 6 [file Image4.PDF]

Supplementary Material

Changes in metabolically active bacterial community during rumen development, and their alteration by rhubarb root powder revealed by 16S rRNA amplicon sequencing

Zuo Wang [1,2,3], Chijioke Elekwachi [3], Jinzhen Jiao [1], Min Wang [1], Shaoxun Tang [1], Chuanshe Zhou [1], Zhiliang Tan [1*], and Robert J. Forster [3*]

[1] Key Laboratory for Agro-Ecological Processes in Subtropical Region, Hunan Research Center of Livestock & Poultry Sciences, South-Central Experimental Station of Animal Nutrition and Feed Science in Ministry of Agriculture, Institute of Subtropical Agriculture, Chinese Academy of Sciences, Changsha, Hunan 410125, China

[2] University of Chinese Academy of Sciences, Beijing 100049, China

[3] Lethbridge Research and Development Centre, Agriculture and Agri-Food Canada, Lethbridge, AB T1J 4B1, Canada

*** Correspondence:**

Zhiliang Tan; Robert J. Forster

zltan@isa.ac.cn; robert.forster@agr.gc.ca

Supplementary Figures

Figure S4. Composition of bacterial communities at phylum level in four fractions of control diet and rhubarb treatment on 50 d (A) and 60 d (B).

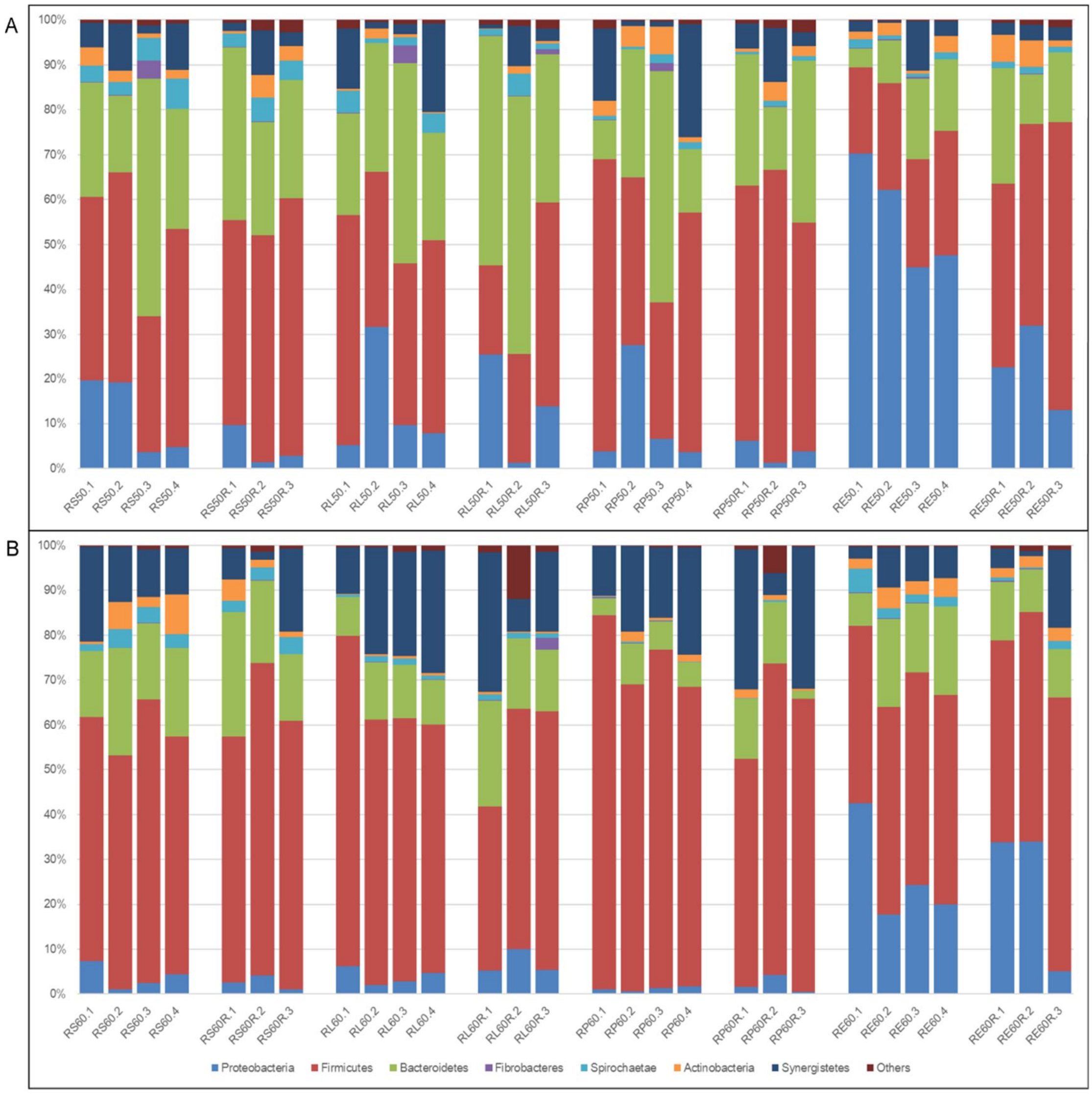
A
B
100%
90%
80%
70%
60%
50%
40%
30%
20%
10%
0%
RS50.1 RS50.2 RS50.3 RS50.4 RS50R.1 RS50R.2 RS50R.3 RL50.1 RL50.2 RL50.3 RL50.4 RL50R.1 RL50R.2 RL50R.3 RP50.1 RP50.2 RP50.3 RP50.4 RP50R.1 RP50R.2 RP50R.3 RE50.1 RE50.2 RE50.3 RE50.4 RE50R.1 RE50R.2 RE50R.3
RS60.1 RS60.2 RS60.3 RS60.4 RS60R.1 RS60R.2 RS60R.3 RL60.1 RL60.2 RL60.3 RL60.4 RL60R.1 RL60R.2 RL60R.3 RP60.1 RP60.2 RP60.3 RP60.4 RP60R.1 RP60R.2 RP60R.3 RE60.1 RE60.2 RE60.3 RE60.4 RE60R.1 RE60R.2 RE60R.3
Proteobacteria
Firmicutes
Bacteroidetes
Fibrobacteres
Spirochaetae
Actinobacteria
Synergistetes
Others
