# Supplementary material for: Changes in Metabolically Active Bacterial Community during Rumen Development, and Their Alteration by Rhubarb Root Powder Revealed by 16S rRNA Amplicon Sequencing
Source: Front Microbiol. 2017 Feb 7;8:159. doi: 10.3389/fmicb.2017.00159 (PMC5293741; doi:10.3389/fmicb.2017.00159)
Supplement: Supplementary file 8 [file Image6.PDF]

Supplementary Material

Changes in metabolically active bacterial community during rumen development, and their alteration by rhubarb root powder revealed by 16S rRNA amplicon sequencing

Zuo Wang [1,2,3], Chijioke Elekwachi [3], Jinzhen Jiao [1], Min Wang [1], Shaoxun Tang [1], Chuanshe Zhou [1], Zhiliang Tan [1*], and Robert J. Forster [3*]

[1] Key Laboratory for Agro-Ecological Processes in Subtropical Region, Hunan Research Center of Livestock & Poultry Sciences, South-Central Experimental Station of Animal Nutrition and Feed Science in Ministry of Agriculture, Institute of Subtropical Agriculture, Chinese Academy of Sciences, Changsha, Hunan 410125, China

[2] University of Chinese Academy of Sciences, Beijing 100049, China

[3] Lethbridge Research and Development Centre, Agriculture and Agri-Food Canada, Lethbridge, AB T1J 4B1, Canada

*** Correspondence:**

Zhiliang Tan; Robert J. Forster

zltan@isa.ac.cn; robert.forster@agr.gc.ca

Supplementary Figures

Figure S6. Composition of bacterial communities at genus level in four fractions of control diet and rhubarb treatment on 50 d (A) and 60 d (B).

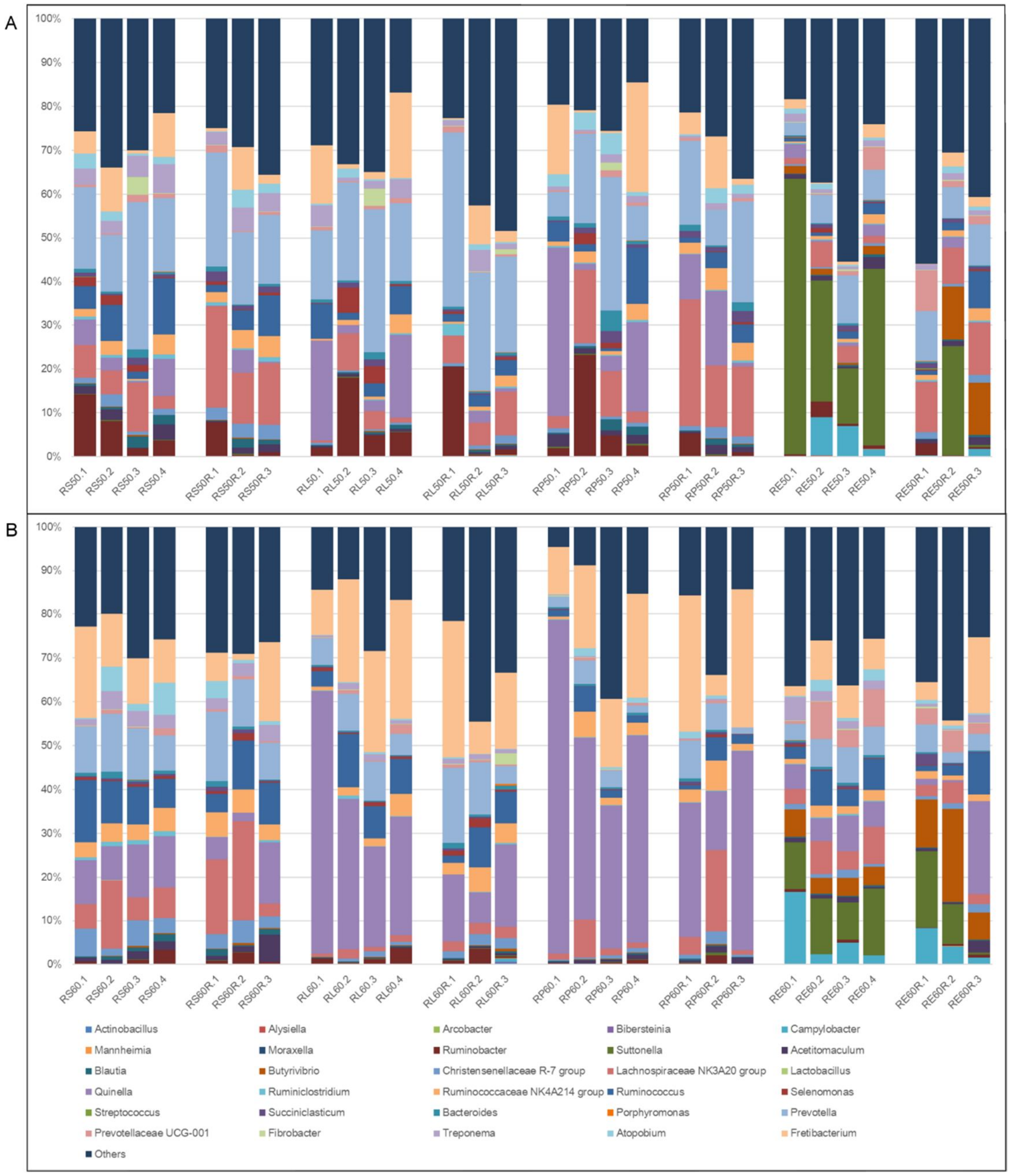
A
B
100%
90%
80%
70%
60%
50%
40%
30%
20%
10%
0%
RS50.1
RS50.2
RS50.3
RS50.4
RS50R.1
RS50R.2
RS50R.3
RL50.1
RL50.2
RL50.3
RL50.4
RL50R.1
RL50R.2
RL50R.3
RP50.1
RP50.2
RP50.3
RP50.4
RP50R.1
RP50R.2
RP50R.3
RE50.1
RE50.2
RE50.3
RE50.4
RE50R.1
RE50R.2
RE50R.3
RS60.1
RS60.2
RS60.3
RS60.4
RS60R.1
RS60R.2
RS60R.3
RL60.1
RL60.2
RL60.3
RL60.4
RL60R.1
RL60R.2
RL60R.3
RP60.1
RP60.2
RP60.3
RP60.4
RP60R.1
RP60R.2
RP60R.3
RE60.1
RE60.2
RE60.3
RE60.4
RE60R.1
RE60R.2
RE60R.3
Actinobacillus
Alysiella
Arcobacter
Bibersteinia
Campylobacter
Mannheimia
Moraxella
Ruminobacter
Suttonella
Acetitomaculum
Blautia
Butyrivibrio
Christensenellaceae R-7 group
Lachnospiraceae NK3A20 group
Lactobacillus
Quinella
Ruminiclostridium
Ruminococcaceae NK4A214 group
Ruminococcus
Selenomonas
Streptococcus
Succiniclasticum
Bacteroides
Porphyromonas
Prevotella
Prevotellaceae UCG-001
Fibrobacter
Treponema
Atopobium
Fretibacterium
Others
