# Supplementary material for: Changes in Metabolically Active Bacterial Community during Rumen Development, and Their Alteration by Rhubarb Root Powder Revealed by 16S rRNA Amplicon Sequencing
Source: Front Microbiol. 2017 Feb 7;8:159. doi: 10.3389/fmicb.2017.00159 (PMC5293741; doi:10.3389/fmicb.2017.00159)
Supplement: Supplementary file 9 [file Image7.PDF]

Supplementary Material

Changes in metabolically active bacterial community during rumen development, and their alteration by rhubarb root powder revealed by 16S rRNA amplicon sequencing

Zuo Wang [1,2,3], Chijioke Elekwachi [3], Jinzhen Jiao [1], Min Wang [1], Shaoxun Tang [1], Chuanshe Zhou [1], Zhiliang Tan [1*], and Robert J. Forster [3*]

[1] Key Laboratory for Agro-Ecological Processes in Subtropical Region, Hunan Research Center of Livestock & Poultry Sciences, South-Central Experimental Station of Animal Nutrition and Feed Science in Ministry of Agriculture, Institute of Subtropical Agriculture, Chinese Academy of Sciences, Changsha, Hunan 410125, China

[2] University of Chinese Academy of Sciences, Beijing 100049, China

[3] Lethbridge Research and Development Centre, Agriculture and Agri-Food Canada, Lethbridge, AB T1J 4B1, Canada

*** Correspondence:**

Zhiliang Tan; Robert J. Forster

zltan@isa.ac.cn; robert.forster@agr.gc.ca

Supplementary Figures

Figure S7. Comparison of Chao 1 index of bacterial communities across different fractions on different days. The horizontal lines in each box indicate the median values, and the 75th and 25th quartile values are respectively represented by the top and bottom sides of each box

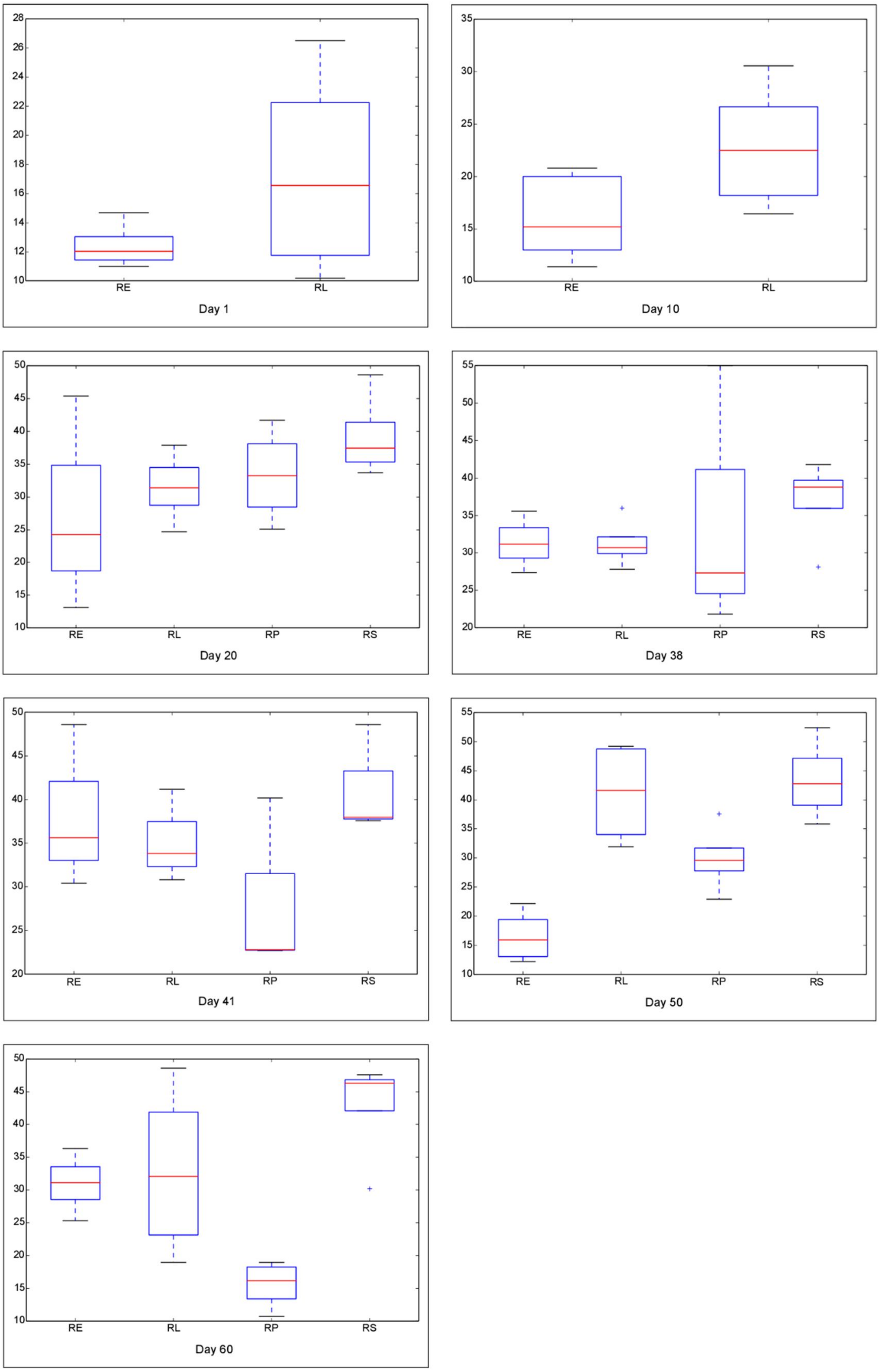
RE
RL
Day 1
RE
RL
Day 10
RE
RL
RP
RS
Day 20
RE
RL
RP
RS
Day 38
RE
RL
RP
RS
Day 41
RE
RL
RP
RS
Day 50
RE
RL
RP
RS
Day 60
