# Supplementary material for: Changes in Metabolically Active Bacterial Community during Rumen Development, and Their Alteration by Rhubarb Root Powder Revealed by 16S rRNA Amplicon Sequencing
Source: Front Microbiol. 2017 Feb 7;8:159. doi: 10.3389/fmicb.2017.00159 (PMC5293741; doi:10.3389/fmicb.2017.00159)
Supplement: Supplementary file 10 [file Image8.PDF]

Supplementary Material

Changes in metabolically active bacterial community during rumen development, and their alteration by rhubarb root powder revealed by 16S rRNA amplicon sequencing

Zuo Wang [1,2,3], Chijioke Elekwachi [3], Jinzhen Jiao [1], Min Wang [1], Shaoxun Tang [1], Chuanshe Zhou [1], Zhiliang Tan [1*], and Robert J. Forster [3*]

[1] Key Laboratory for Agro-Ecological Processes in Subtropical Region, Hunan Research Center of Livestock & Poultry Sciences, South-Central Experimental Station of Animal Nutrition and Feed Science in Ministry of Agriculture, Institute of Subtropical Agriculture, Chinese Academy of Sciences, Changsha, Hunan 410125, China

[2] University of Chinese Academy of Sciences, Beijing 100049, China

[3] Lethbridge Research and Development Centre, Agriculture and Agri-Food Canada, Lethbridge, AB T1J 4B1, Canada

*** Correspondence:**

Zhiliang Tan; Robert J. Forster

zltan@isa.ac.cn; robert.forster@agr.gc.ca

Supplementary Figures

Figure S8. Principal coordinate analysis (PCoA) of bacterial community structure using unweighted Unifrac matrix within different fractions on different days

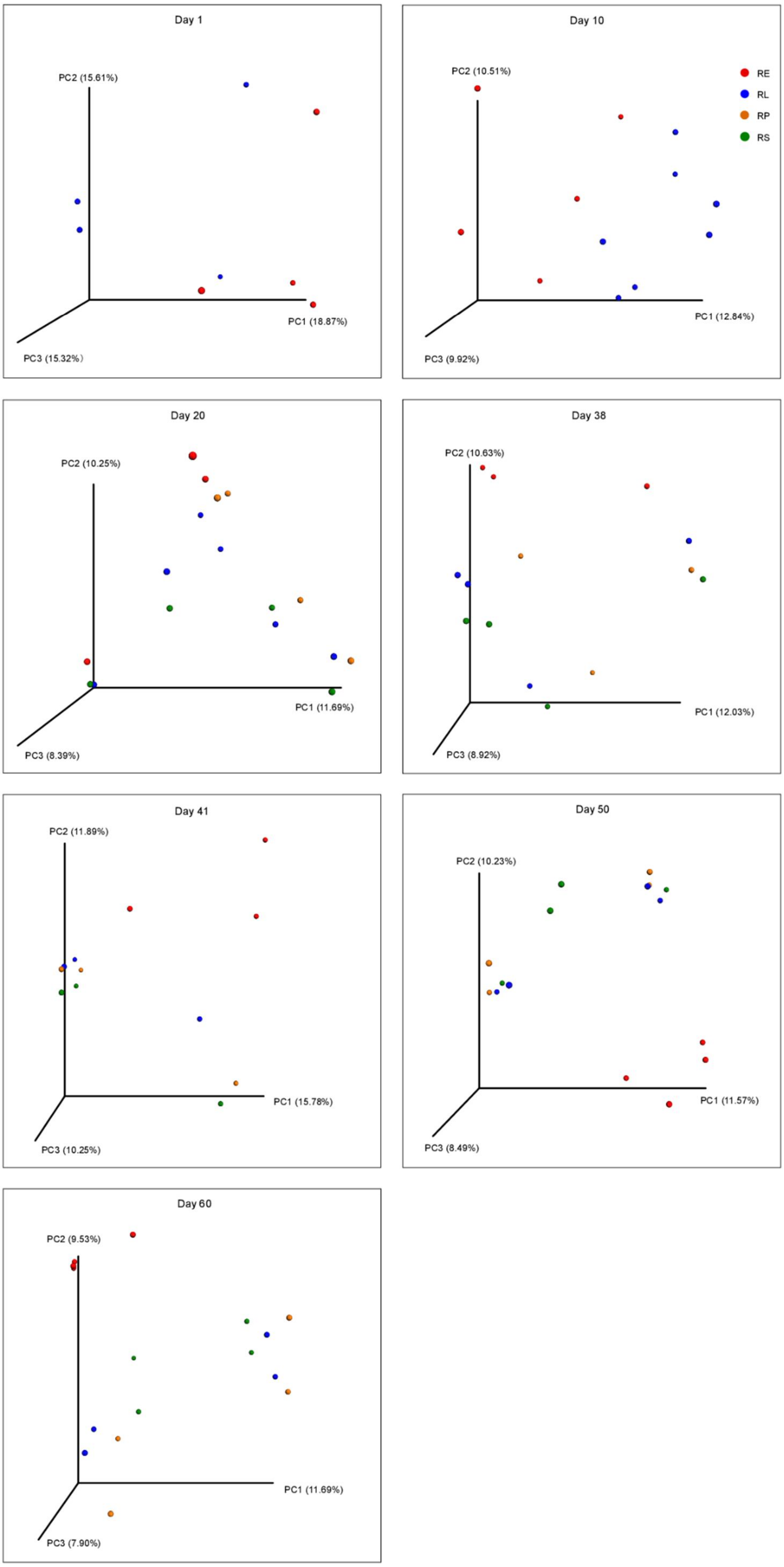
Day 1
PC2 (15.61%)
PC1 (18.87%)
PC3 (15.32%)
Day 10
PC2 (10.51%)
PC1 (12.84%)
PC3 (9.92%)
RE
RL
RP
RS
Day 20
PC2 (10.25%)
PC1 (11.69%)
PC3 (8.39%)
Day 38
PC2 (10.63%)
PC1 (12.03%)
PC3 (8.92%)
Day 41
PC2 (11.89%)
PC1 (15.78%)
PC3 (10.25%)
Day 50
PC2 (10.23%)
PC1 (11.57%)
PC3 (8.49%)
Day 60
PC2 (9.53%)
PC1 (11.69%)
PC3 (7.90%)
